# Cardiomyocyte-derived OTUD7B promotes cardiac hypertrophy by deubiquitinating SERCA2a

**DOI:** 10.7150/thno.129105

**Published:** 2026-02-04

**Authors:** Zhuqi Huang, Xue Han, Yuxing Hou, Xingchen Wang, Fuyu Qiu, Yingchao Gong, Nipon Chattipakorn, Guosheng Fu, Guang Liang, Dongwu Lai

**Affiliations:** 1 Department of Cardiology, Zhejiang Key Laboratory of Cardiovascular Intervention and Precision Medicine, Sir Run Run Shaw Hospital, Zhejiang University School of Medicine, Hangzhou, Zhejiang, 310020, China.; 2 Department of Pharmacy and Institute of Inflammation, Zhejiang Provincial People's Hospital, Affiliated People's Hospital, Hangzhou Medical College, Hangzhou, Zhejiang, 310014, China.; 3 Zhejiang-Thailand Joint Lab of Cardiovascular Diseases, School of Pharmaceutical Sciences, Hangzhou Medical College, Hangzhou, Zhejiang, 311399, China.; 4 Department of Cardiology, the First Affiliated Hospital, Wenzhou Medical University, Wenzhou, Zhejiang, 325035, China.; 5 Cardiac Electrophysiology Research and Training Center, Faculty of Medicine, Chiang Mai University, Chiang Mai, 50200, Thailand.

**Keywords:** deubiquitinating enzyme, OTUD7B, cardiomyocyte, cardiac hypertrophy, SERCA2a

## Abstract

**Rationale:** Pathological cardiac hypertrophy, triggered by persistent neurohumoral or hemodynamic stress, is a key precursor of ventricular dysfunction and heart failure. Deubiquitinating enzymes (DUBs) have emerged as critical regulators of cardiovascular biology. This study examined the function of a DUB, ovarian tumor domain-containing 7B (OTUD7B), in cardiac hypertrophy.

**Methods:** Cardiomyocyte-specific OTUD7B knockout and overexpression mouse models were generated to evaluate myocardial hypertrophy and cardiac dysfunction in response to angiotensin II (Ang II) infusion or transverse aortic constriction (TAC). Quantitative ubiquitinome analysis, site-directed mutagenesis, and co-immunoprecipitation assays were performed to explore the substrate and mechanism of OTUD7B.

**Results:** Transcriptomic and experimental validation demonstrated that cardiomyocyte OTUD7B was increased in hypertrophic hearts of both humans and mice. Cardiomyocyte-specific deletion of OTUD7B significantly mitigated angiotensin II (Ang II)- and transverse aortic constriction (TAC)-induced cardiac hypertrophy and dysfunction in mice. Mechanistically, quantitative ubiquitinome analysis identified sarcoplasmic/endoplasmic reticulum Ca^2+^ ATPase 2a (SERCA2a) as a direct substrate of OTUD7B. OTUD7B bound to SERCA2a and removed K63-linked ubiquitin at K628 through its catalytic site C194. This deubiquitination promoted SERCA2a-phospholamban (PLN) interaction, thereby restricting SERCA2a activity in Ca²⁺ handling and driving hypertrophic response in cardiomyocytes. Moreover, cardiomyocyte-specific OTUD7B overexpression exacerbated TAC-induced cardiac hypertrophy and dysfunction by deubiquitinating SERCA2a at K628.

**Conclusions:** This study defines a novel OTUD7B-SERCA2a regulatory axis and identifies OTUD7B as a promising therapeutic target for cardiac hypertrophy and dysfunction.

## Introduction

Cardiac hypertrophy is defined as a typical compensatory adaptation to sustained hemodynamic or neurohumoral stimulation, including hypertension, aortic stenosis, or persistent neuroendocrine activation 1. Although initially beneficial for maintaining cardiac output, prolonged hypertrophic remodelling often evolves into contractile impairment, ventricular dilation, and ultimately heart failure 2. At the cellular level, pathological hypertrophy is characterized by enlarged cardiomyocytes, fetal gene reactivation, and progressive interstitial fibrosis 3. While pharmacological therapies such as β-adrenergic antagonists and angiotensin-converting enzyme (ACE) inhibitors provide clinical benefit, their capacity to reverse established hypertrophy remains limited 4. Consequently, elucidating the molecular mechanisms governing this maladaptive process and uncovering new intracellular therapeutic targets remains of great importance.

Ubiquitination, a predominant post-translational modification, has been demonstrated to play a critical role in the modulation of protein turnover, localization, and activity 5. This reversible process is controlled by the coordinated actions of ubiquitin ligases (E3s), which conjugate ubiquitin chains to substrates, and deubiquitinating enzymes (DUBs), which remove them 6. Ubiquitin linkage patterns-including M1, K6, K11, K27, K29, K33, K48, and K63-determine distinct functional outcomes for substrate proteins 7. DUBs thus serve as pivotal regulators of protein fate, and accumulating evidence implicates them in diverse cardiovascular disorders such as myocardial infarction 8, atherosclerosis 9, and cardiac hypertrophy 10, 11. Accordingly, targeting specific DUBs could provide promising therapeutic strategies for cardiac hypertrophy.

Our multiple transcriptomic profiling identified ovarian tumor domain-containing protein 7B (OTUD7B), a member of the ovarian tumor (OTU) family of DUBs 12, as a candidate modulator of myocardial hypertrophic remodeling. OTUD7B was initially characterized as a regulator of the non-canonical NF-κB pathway by catalyzing the removal of ubiquitin chains from TRAF3 12. Recent studies have revealed that OTUD7B functions as a cell-cycle-regulated deubiquitinase that antagonizes APC/C-mediated K11-linked ubiquitination of mitotic substrates 13. Furthermore, OTUD7B has been reported to deubiquitinate LSD1, thereby modulating LSD1 stability and breast cancer metastasis 14. Considering the growing recognition of DUB-mediated post-translational regulation in cardiac remodelling, it is of considerable interest to ascertain whether OTUD7B modulates critical cardiac proteins, thereby influencing cardiac hypertrophy.

This study aimed to delineate the role and underlying mechanism of OTUD7B in pathological cardiac hypertrophy. We demonstrated that cardiomyocyte-derived OTUD7B was increased in hypertrophic myocardium from both mouse and human. Deletion of OTUD7B specifically in cardiomyocytes mitigated Ang II- or TAC-induced cardiac hypertrophy and dysfunction. Mechanistically, OTUD7B removed K63-linked ubiquitin from sarcoplasmic/endoplasmic reticulum Ca^2+^ ATPase 2a (SERCA2a) at K628 through its catalytic site C194, thereby enhancing SERCA2a-phospholamban (PLN) interaction and constraining SERCA2a activity in cardiomyocytes. These findings reveal a novel OTUD7B-SERCA2a axis that governs cardiomyocyte hypertrophy and highlight OTUD7B as a promising therapeutic target for pathological cardiac hypertrophy and dysfunction.

## Materials and Methods

### Reagents

Primary antibodies against GAPDH (5174) and PLN (14562) were obtained from Cell Signaling Technology (Danvers, MA, USA). F4/80 (ab6640), SERCA2a (ab3625), and α-actinin (ab137346) antibodies were sourced from Abcam (Cambridge, UK), while OTUD7B (sc-514402) was purchased from Santa Cruz Biotechnology (Santa Cruz, CA, USA). Proteintech (Wuhan, China) supplied antibodies for Flag (20543-1-AP, 66008-4-Ig), HA (51064-2-AP, 66006-2-Ig), Myc (60003-2-Ig, 16286-1-AP), and OTUD7B (16605-1-AP). Vimentin (bs-0756R) was acquired from Bioss (Beijing, China), and MYH7 (A7564) and ANP (A1609) were purchased from ABclonal (Wuhan, China). Angiotensin II (Ang II; HY-13948) and tamoxifen (HY-13757A) were obtained from MedChemExpress (New Jersey, USA).

### *In vivo* randomization and blinding procedures

Sample size determination was conducted in advance using G-Power version 3.1.9, assuming a power of 0.80 and a two-sided significance level of 0.05, resulting in six mice per experimental group. Allocation of animals was carried out using a randomization procedure based on a random number table. All *in vivo* experiments were performed under blinded conditions. Specifically, mice were first assigned temporary random identifiers within comparable body-weight ranges and then randomly allocated to the respective experimental groups, after which permanent cage numbers were assigned. Cages for each experimental group were randomly chosen from the complete pool of available cages. Data collection and all subsequent analyses were conducted independently by two investigators blinded to both the treatment and group allocation.

### Human heart samples

Human myocardial specimens were collected from individuals diagnosed with cardiac hypertrophy at the time of pacemaker lead replacement surgery. Control myocardial specimens without hypertrophic features were collected from individuals diagnosed with arrhythmias who underwent the same surgical intervention and exhibited no clinical or pathological signs of cardiac hypertrophy. All participants provided written informed consent prior to inclusion. The experimental procedures were examined and authorized by the Institutional Ethics Committee of The First Affiliated Hospital of Wenzhou Medical University (approval number KY2022-156) and was conducted in accordance with the ethical standards set forth in the Declaration of Helsinki. The clinical characteristics of the human subjects are summarized in Table S1.

### Animal experiments

All animal handling and experimental protocols were reviewed and authorized by the Animal Policy and Welfare Committee of Hangzhou Medical College (approval number ZJCLA-IACUC-20030068). All procedures were conducted in strict accordance with the National Institutes of Health Guide for the Care and Use of Laboratory Animals. OTUD7B^flox^/^flox^ (OTUD7B^f/f^) mice, and cardiomyocyte-specific OTUD7B deficient mice (OTUD7B^f/f^+Myh6-CreERT2^+/-^, OTUD7B^CKO^) were provided by GemPharmatech (Nanjing, China). Genotyping was performed by PCR using the primer sequences listed in Table S2. Only male mice were included in the present study. Animals were maintained under controlled conditions with a 12 h light/12 h dark cycle, stable ambient temperature, and ad libitum access to a standard laboratory chow. All mice were allowed to acclimate to the animal facility for a minimum of two weeks prior to experimental manipulation. Both experimental procedures and data analysis were conducted in a blinded manner. Male mice were deliberately chosen, as previous studies have indicated that female mice may develop mild cardiac fibrosis and cardiac dysfunction influenced by estrogen signaling and relatively lower testosterone levels 15.

(1) Ang II-induced hypertrophy model: Six-week-old male OTUD7B^f/f^ and OTUD7B^CKO^ mice received intraperitoneal tamoxifen injections for 7 consecutive days. After a 1-week period, mice were infused with Ang II (1 μg/kg/min) or saline for 4 weeks using a subcutaneously implanted micro-osmotic pump (Alzet, CA, USA). Systolic blood pressure (SBP) in conscious animals was monitored weekly using a noninvasive tail-cuff system (BP-2010A, Softron, Japan).

(2) Transverse aortic constriction (TAC)-induced hypertrophy model: Six-week-old male OTUD7B^f/f^ mice and OTUD7B^CKO^ mice were administered tamoxifen intraperitoneally for 1 week, followed by a 1-week interval. Mice were then anesthetized with isoflurane and subjected to TAC surgery. After partial thoracotomy, the aortic arch was exposed and constricted using a 27-gauge needle and a 6-0 nylon suture. Sham-operated mice underwent identical surgical procedures without ligation. Animals were maintained for 4 weeks after TAC or sham surgery.

(3) OTUD7B (OTUD7B^OE^) and SERCA2a (SERCA2a^WT^ or SERCA2a^K628R^) cardiomyocyte-specific overexpression was achieved using recombinant adeno-associated virus serotype 9 (AAV9) vectors driven by the cTNT promoter (Genechem, Shanghai, China). Male wild-type mice at 6 weeks of age received tail-vein injections of AAV9 (2×10^11^ viral particles per mouse). Two weeks after viral delivery, mice underwent TAC surgery and were followed for an additional 4 weeks.

Transthoracic echocardiography was performed under isoflurane anesthesia, during which left ventricular short-axis dimensions and the transmitral E/A ratio were acquired using a high-frequency ultrasound platform (Vevo 3100; VisualSonics, Japan).

At the study endpoint, mice were euthanized under sodium pentobarbital anesthesia, and blood and cardiac tissues were harvested for subsequent analyses.

### Immunostaining and histological analysis

For histological evaluation, paraffin-embedded cardiac tissues were sectioned at a thickness of 5 μm and stained with hematoxylin and eosin (HE; G1120, Solarbio). Cardiomyocyte cross-sectional area was assessed using OCT-embedded tissue sections labeled with wheat germ agglutinin (WGA). Myocardial fibrosis was examined on paraffin sections following Masson's trichrome staining (G1340, Solarbio). Histological images were acquired using either a bright-field or fluorescence microscope (Nikon 80i).

To determine the cellular sources of OTUD7B expression in cardiac tissue, 5 μm OCT-embedded sections were fixed in 4% formaldehyde for 10 min, permeabilized with 0.1% Triton X-100 for 10 min, and blocked with 5% bovine serum albumin for 30 min at room temperature. Sections were subsequently incubated overnight at 4 °C with primary antibodies against OTUD7B, α-actinin, F4/80, and vimentin. After incubation with fluorophore-labeled secondary antibodies, nuclei were counterstained with DAPI. Confocal images were captured using a laser scanning confocal microscope (Nikon A1).

### Cell culture and transfection

Human embryonic kidney 293T cells (GNHu17) were obtained from the Shanghai Institute of Biochemistry and Cell Biology. Neonatal rat cardiomyocytes (NRCMs) and neonatal rat cardiac fibroblasts (NRCFs) were isolated from the ventricles of neonatal Sprague-Dawley rats according to previously published protocols 16, 17. Mouse primary peritoneal macrophages (MPMs) were isolated following the protocols described in previous reports 18. All cell types were maintained in a humidified incubator at 37 °C with 5% CO₂. 293T cells, NRCMs, and NRCFs were cultured in high-glucose Dulbecco's modified Eagle's medium (DMEM; Gibco, Eggenstein, Germany) with 10% fetal bovine serum (FBS, BC-SE-FBS01, BioChannel, China) and 1% penicillin/streptomycin (Invitrogen, Waltham, MA, USA). MPMs were cultured in RPMI-1640 medium (Gibco) containing 10% FBS and 1% penicillin/streptomycin. The final concentration of Ang II in the culture medium was 1 μM. Adult mouse cardiomyocytes (AMCMs) were isolated from OTUD7B^f/f^ and OTUD7B^CKO^ mouse hearts using a simplified Langendorff-free isolation protocol 19.

Gene knockdown and ectopic expression in cultured cells were accomplished through transient transfection of sequence-specific siRNAs or expression plasmids. Custom-designed siRNAs were constructed by Genepharma (Shanghai, China) for rat OTUD7B (CCUCAGUGACUUUGAACAATT) and SERCA2a (CUGCGAACUUCAUCAAAUATT). Plasmids encoding Flag-OTUD7B^WT^, Flag-OTUD7B^C194A^, OTUD7B^H358A^, Myc-SERCA2a^WT^, Myc-SERCA2a^K611R^, Myc-SERCA2a^K628R^, HA-Ub, HA-Ub-K48, and HA-Ub-K63 were generated by GENEWIZ (Suzhou, China). NRCMs were transfected with siRNAs using Lipofectamine™ 2000 (Thermo Fisher Scientific, Carlsbad, CA, USA), while plasmid transfections in NRCMs and 293T cells were carried out with Lipofectamine™ 3000 (Thermo Fisher Scientific) following the manufacturer's protocols.

### Western blotting and co-immunoprecipitation

Protein lysates were prepared from cultured cells and myocardial samples using a commercially available extraction reagent (AR0103, Boster, Wuhan, China). The resulting protein samples were resolved by electrophoresis on 10% polyacrylamide gels under denaturing conditions and then electroblotted onto polyvinylidene difluoride membranes. Membranes were blocked for 1.5 h at room temperature in Tris-buffered saline (pH 7.4) containing 0.1 % Tween-20 and 5 % non-fat milk prior to incubation with primary antibodies. Immunoreactive signals were visualized using secondary antibodies coupled to horseradish peroxidase in combination with a chemiluminescent substrate. Signal acquisition was optimized to avoid saturation and to maintain measurements within a linear response window. Densitometric analysis of immunoblots was conducted using ImageJ software (NIH, MD, USA), with protein levels expressed relative to the corresponding internal references.

For protein interaction analyses, lysates obtained from treated cells were subjected to immunoaffinity enrichment by incubation with the indicated primary antibodies at 4 °C overnight. Antibody-protein complexes were subsequently isolated by incubation with protein A/G-conjugated agarose beads (P2012; Beyotime, Shanghai, China) for 2 h at 4 °C. The recovered complexes were resolved by immunoblotting to identify associated proteins, while aliquots of whole-cell extracts were processed in parallel as input references. Interaction-associated signal intensities were determined by densitometric evaluation using ImageJ.

### Real-time quantitative PCR

RNA from cultured cells and heart tissue was prepared using RNAiso Plus (Takara, Japan). First-strand cDNA was then generated with the PrimeScript™ RT Reagent Kit (Takara), following the instructions provided by the manufacturer. Gene expression analysis was carried out via quantitative real-time PCR on a QuantStudio™ 3 system (Thermo Fisher Scientific). Primer sequences were listed in Table S3.

### Transcriptome analysis

Two public mouse datasets (GSE221396 20, GSE284286 21) and two public human datasets (GSE71613 22, GSE89714 23) were obtained from the Gene Expression Omnibus repository. In GSE221396, mice were challenged with Ang II for 4 weeks and heart tissues were acquired. In GSE284286, mice were challenged with Ang II for 3 weeks and heart tissues were acquired. In GSE71613, human cardiac tissues were acquired from patients with heart failure and donor controls. In GSE89714, human cardiac tissues were acquired from patients with hypertrophic cardiomyopathy and donor controls. Differentially expressed genes (DEGs) were selected with log2(fold change) >0.3 or log2(fold change) <-0.3 and *P*-value < 0.05 using GEO2R.

### Single-cell RNA sequencing analysis

Single-cell RNA sequencing analysis was conducted on the hearts of mice subjected to TAC treatment from one publicly available mouse dataset (GSE120064 24). Single-cell transcriptional profiles were processed using the Seurat package (version 4.0, https://satijalab.org/seurat/). Cells were organized in low-dimensional space by Uniform Manifold Approximation and Projection to define discrete clusters. Cell types were subsequently assigned through manual curation according to established marker gene expression patterns.

### Quantitative ubiquitinome

Cell samples were disrupted using an appropriate protein extraction buffer (8 M urea, 1% protease inhibitor cocktail). The supernatant was collected, and the protein concentration was determined with the BCA kit according to the manufacturer's instructions. The peptides were obtained by digesting the protein with trypsin. The resulting peptide mixtures were subjected to ubiquitin remnant enrichment using antibody-conjugated beads that had been pre-equilibrated (PTM Bio, Hangzhou, China). Comprehensive quantitative profiling of ubiquitinated peptides was subsequently performed by PTM Bio. The KEGG analysis was performed among proteins with reduced ubiquitination modification in the OTUD7B^WT^ group compared to empty vector (EV) group. The raw data were deposited in the ProteomeXchange repository (PXD068918).

### Intracellular Ca^2+^ detection

AMCMs or NRCMs were incubated with Fluo-4 AM at a final concentration of 2 μM and incubated in a 37℃ incubator for twenty minutes for fluorescence probe loading. Washed with PBS twice, cells were placed in a spinning-disk confocal microscope (SpinSR, Olympus, Japan) to detect the transient change of intracellular Ca^2+^ concentration by measuring fluorescence when AMCMs were exposed to 10 mM caffeine.

### Ca^2+^ transient measurement

Ca^2+^ transients were recorded in AMCMs under electrical pacing (1 Hz) by MyoPacer EP Cell Stimulator (IonOptix, MA, USA) and measured by loading cells with Fura-2-AM (2 µM) at 37 °C for 20 min. The cells were washed three times with Tyrode's solution. Fluorescence signals were detected by an IonOptix system (IonOptix).

### Assessment of cardiomyocyte contractility

AMCMs were paced electrically at 1 Hz to achieve stable rhythmic contractions. Changes in cell length during relaxation and contraction were captured using an IonOptix recording platform. Fractional shortening was expressed as the percentage reduction in cell length, calculated from the difference between resting and contracted states normalized to the resting length.

### Statistical analysis

Data acquisition and analysis were independently conducted by two investigators blinded to both experimental allocation and treatment conditions. Animal studies were performed with six independent biological samples per group, whereas cellular assays included three independent biological replicates. Results are presented as mean ± standard error of the mean (SEM). Sample sizes for individual experiments are specified, with “n” denoting biological rather than technical replication. For cell-based assays, each data point reflected an aggregate measurement from multiple cells; therefore, normality was assumed in accordance with the central limit theorem. In animal experiments, where group sizes were six, data distributions were formally assessed using the Shapiro-Wilk test, with *P* values greater than 0.05 indicating no significant deviation from normality. Statistical comparisons between two groups were carried out using unpaired two-sided Student's t-tests. When more than two groups were compared, one-way analysis of variance followed by Tukey's multiple-comparison procedure was applied. Experiments involving repeated measurements from the same subject were evaluated using two-way repeated-measures ANOVA with a pooled variance and Tukey-adjusted post hoc testing. A threshold of *P* < 0.05 was considered indicative of statistical significance. Post hoc analyses were performed only when the overall test reached significance and variance homogeneity assumptions were met. All statistical evaluations were completed using GraphPad Prism software (version 8.0; GraphPad, CA, USA).

## Results

### Upregulation of cardiomyocyte OTUD7B in hypertrophic hearts

**Figure 1:**
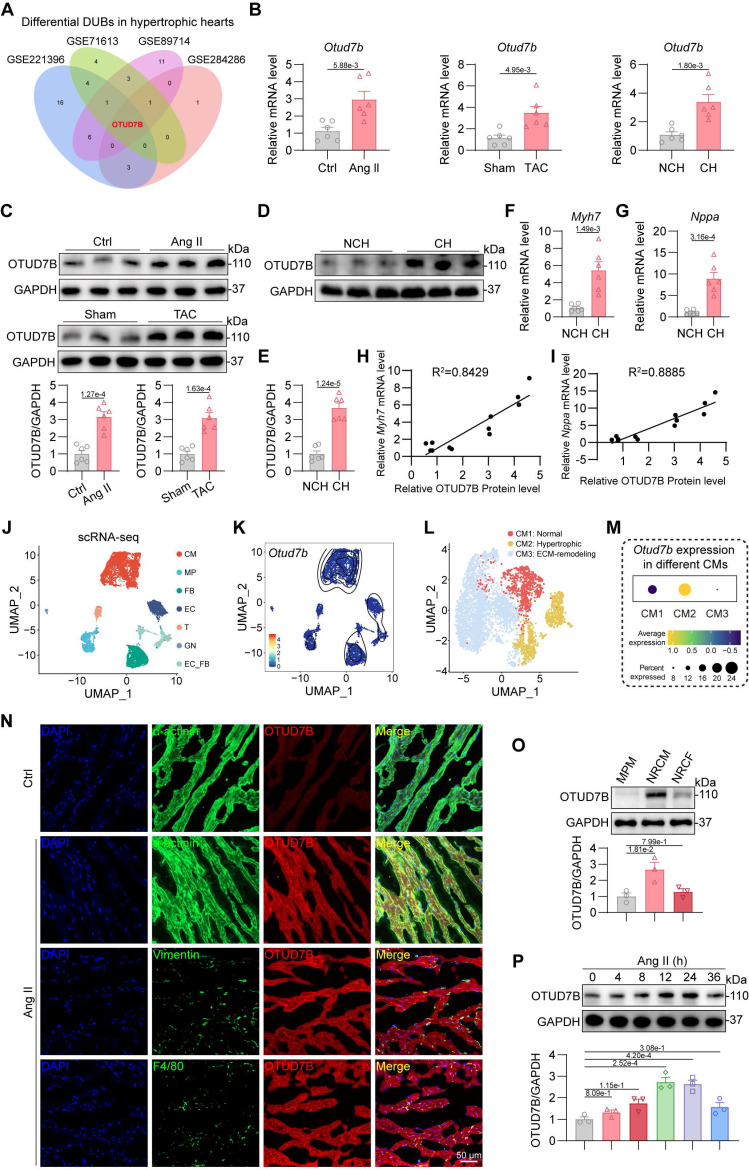
** Upregulation of cardiomyocyte OTUD7B in hypertrophic hearts.** (A) Transcriptome analysis was performed to analyze the expression profile of deubiquitinating enzymes (DUBs) in hypertrophic hearts from two public mouse datasets (GSE221396, GSE284286) and two public human datasets (GSE71613, GSE89714). Venn diagram representing differentially expressed genes in hypertrophic hearts compared to control hearts. (B) mRNA levels of *Otud7b* in myocardial tissues from mice subjected to Ang II or TAC-induced hypertrophy and patients with cardiac hypertrophy (n = 6). Signal intensities were adjusted using *Actb* as the normalization control. NCH, non-cardiac hypertrophy; CH, cardiac hypertrophy. (C) Western blotting detection and quantitative analysis of OTUD7B in cardiac tissues from mice subjected to Ang II infusion or TAC. GAPDH served as the internal loading reference (n = 6). (D-E) Western blotting detection (D) and quantitative analysis (E) of OTUD7B in heart tissues of patients with cardiac hypertrophy. GAPDH served as the internal loading reference (n = 6). (F-G) Transcript levels of *Myh7* (F) and *Nppa* (G) in myocardial samples from patients exhibiting cardiac hypertrophy (n = 6). Signal intensities were adjusted using *Actb* as the normalization control. (H-I) Pearson correlation coefficients between OTUD7B protein level and *Myh7* (H) or *Nppa* (I) mRNA levels in human hearts. (J) Single-cell mRNA sequencing analysis was conducted on the hearts of mice subjected to TAC treatment from one public mouse dataset (GSE120064). The UMAP visualization delineated seven major cellular populations, comprising cardiomyocytes (CM), macrophages (MP), fibroblasts (FB), endothelial cells (EC), T-lymphocytes (T), granulocytes (GN), and endothelial-fibroblast transitional cells (EC_FB). (K) A two-dimensional scatter analysis illustrating *Otud7b* expression across the identified cell populations. (L) The UMAP visualization resolved cardiomyocytes into three discrete clusters with distinct functional characteristics. (M) A dot-based summary depicted the comparative abundance of *Otud7b* transcripts among the cardiomyocyte subpopulations. (N) Representative immunofluorescence staining of α-actinin (green) or Vimentin (green), or F4/80 (green) and OTUD7B (red) in heart tissues. (O) Western blotting detection and quantitative analysis of OTUD7B in mouse primary peritoneal macrophage (MPM), neonatal rat cardiomyocyte (NRCM), and neonatal rat cardiac fibroblast (NRCF). GAPDH served as the internal loading reference (n = 3). (P) Temporal profiling of OTUD7B expression following Ang II stimulation in neonatal rat cardiomyocytes (NRCMs). Western blotting detection and quantitative analysis of OTUD7B. GAPDH served as the internal loading reference (n = 3).

To identify DUBs implicated in cardiac hypertrophy, we first examined two public mouse datasets (GSE221396 20, GSE284286 21) of hearts from angiotensin II (Ang II)-induced mice and two human datasets (GSE71613 22, GSE89714 23) of human hypertrophic heart tissues. Among the DUB family members, *Otud7b* emerged as the only one changed transcript in hypertrophic hearts compared with controls in all four datasets (Figure 1A). This transcriptional upregulation was consistently reproduced in myocardial tissues from mice subjected to Ang II infusion or transverse aortic constriction (TAC) as well as patients with cardiac hypertrophy (Figure 1B). Western blotting confirmed that OTUD7B protein levels were markedly elevated in the hearts of both Ang II- and TAC-challenged mice (Figure 1C). Consistent with these observations, hypertrophic myocardial specimens from patients exhibited elevated OTUD7B protein levels (Figure 1D-E), accompanied by elevated expression of hypertrophy-associated markers *Myh7* and *Nppa* (Figure 1F-G). Correlation analyses further demonstrated strong positive associations between OTUD7B and these canonical hypertrophic genes (Figure 1H-I), implicating OTUD7B as a molecular feature of pathological cardiac hypertrophy.

In order to localize the cellular source of OTUD7B in hypertrophic hearts, single-cell RNA sequencing (scRNA-seq) analysis was conducted on the hearts of mice subjected to TAC treatment from one publicly available mouse dataset (GSE120064 24). 7 main cell types were classified from scRNA-seq data based on the specific gene markers, including cardiomyocytes (CM), macrophages (MP), fibroblasts (FB), endothelial cells (EC), T-lymphocytes (T), granulocytes (GN), and endothelial-fibroblast transitional cells (EC_FB) (Figure 1J). Single-cell transcriptomic analysis revealed that *Otud7b* transcripts were largely confined to the cardiomyocyte population (Figure 1K). Based on the expression patterns of established marker genes, cardiomyocytes were further stratified into three distinct phenotypic subsets corresponding to normal (CM1), hypertrophic (CM2), and extracellular matrix (ECM)-remodeling (CM3) cardiomyocytes (Figure 1L). Among these subsets, *Otud7b* expression was markedly enriched within the hypertrophic cardiomyocytes (Figure 1M), supporting a putative involvement of OTUD7B in the development of cardiac hypertrophy. Then, double immunofluorescence staining was performed on Ang II-treated mouse hearts. OTUD7B was predominantly found within α-actinin⁺ cardiomyocytes, with minimal overlap in vimentin⁺ fibroblasts or CD68⁺ macrophages (Figure 1N). Immunofluorescence staining also revealed that Ang II stimulation significantly augmented OTUD7B abundance in hearts compared to the control. Protein profiling of mouse primary peritoneal macrophage (MPM), neonatal rat cardiomyocyte (NRCM), and neonatal rat cardiac fibroblast (NRCF) further demonstrated that basal OTUD7B expression was highest in cardiomyocytes (Figure 1O). Moreover, OTUD7B expression in NRCMs upon Ang II stimulation changed in a time-dependent manner (Figure 1P). These data suggest cardiomyocyte OTUD7B as a candidate regulator of cardiac hypertrophy.

### Cardiomyocyte-specific OTUD7B deletion attenuates Ang II-induced cardiac hypertrophy

**Figure 2:**
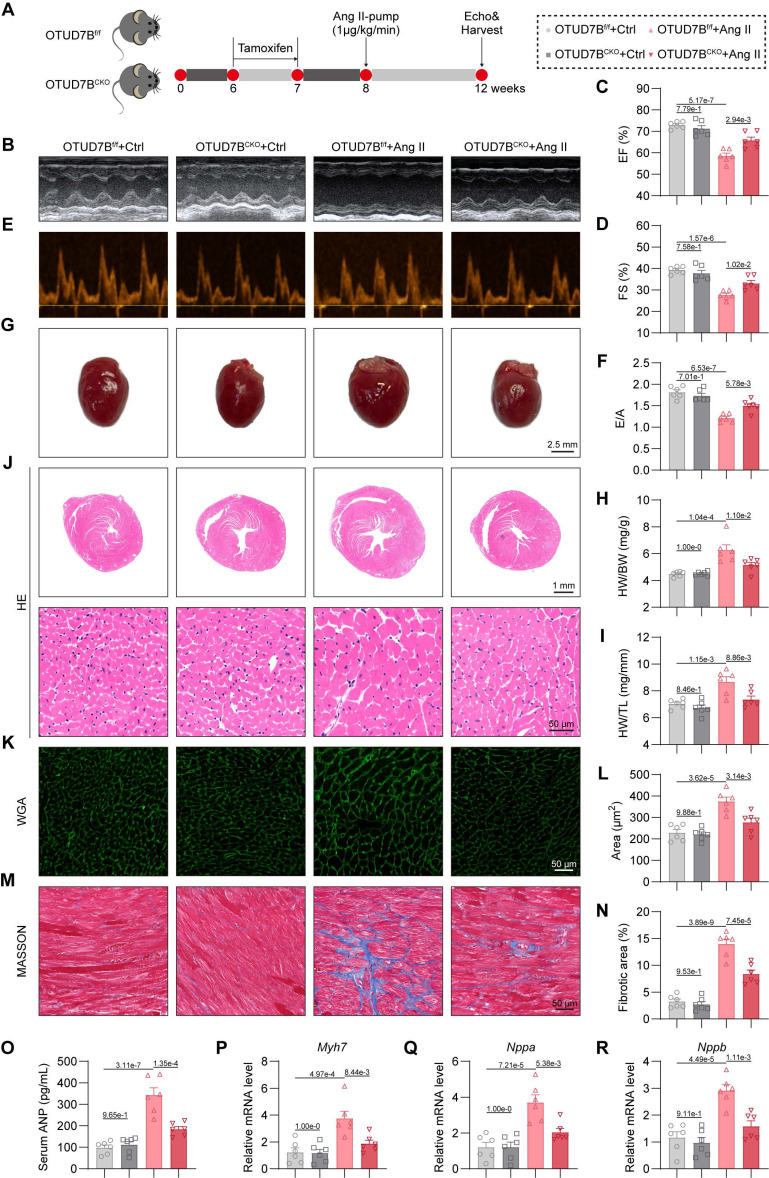
** Cardiomyocyte-specific OTUD7B deletion attenuates Ang II-induced cardiac hypertrophy.** (A) Schematic diagram of the Ang II-induced mouse model. OTUD7B^f/f^ mice and OTUD7B^CKO^ mice were intraperitoneally injected with tamoxifen for 1 week. Another week later, mice were injected with Ang II (1 μg/kg/min) or saline via osmotic pumps for 4 weeks. After 4 weeks, the cardiac function of mice was assessed using echocardiography. Mice were then euthanized, and samples were harvested. (B) Representative left ventricular M-mode echocardiographic images. (C-D) Values of ejection fraction (C) and fractional shortening (D). EF, ejection fraction; FS, fractional shortening; n = 6. (E-F) Representative left ventricular PW Doppler echocardiographic images (E) and values of E/A (F). n = 6. (G) Representative whole heart images. (H) The ratio of heart weight to body weight (HW/BW). n = 6. (I) The ratio of heart weight to tibial length (HW/TL). n = 6. (J) Representative HE-stained images of cardiac tissue sections. (K-L) Representative images (K) and quantification (L) of wheat germ agglutinin (WGA)- stained cardiac tissue sections (n = 6). (M-N) Representative images (M) and quantification (N) from Masson's trichrome-stained cardiac tissue sections (n = 6). (O) Serum atrial natriuretic peptide (ANP) levels were detected using ELISA kits (n = 6). (P-R) mRNA levels of *Myh7* (P), *Nppa* (Q), and *Nppb* (R) in heart tissues (n = 6). Signal intensities were adjusted using *Actb* as the normalization control.

To ascertain the functional relevance of OTUD7B, we generated cardiomyocyte-specific OTUD7B knockout mice (OTUD7B^CKO^) and littermate control mice (OTUD7B^f/f^) (Figure S1). Both groups were subjected to continuous Ang II infusion via osmotic mini-pumps for four weeks (Figure 2A). Cardiomyocyte-targeted deletion of OTUD7B did not alter Ang II-driven elevations in systolic blood pressure (SBP, Figure S2A-B) or serum Ang II levels (Figure S2C). The echocardiographic assessment revealed that OTUD7B^CKO^ mice were resistant to Ang II-induced ventricular dysfunction (Figure 2B-F and Table S4). Morphological assessment confirmed that loss of OTUD7B blunted Ang II-induced cardiac enlargement (Figure 2G), with significant reductions in heart weight normalized to body weight or tibial length (Figure 2H-I). Histological evaluation further demonstrated that OTUD7B^CKO^ mice exhibited attenuated cardiomyocyte enlargement as evidenced by hematoxylin and eosin (HE) and wheat germ agglutinin (WGA) staining (Figure 2J-L). Fibrotic remodeling was also reduced, as demonstrated by Masson's trichrome histological analysis (Figure 2M-N). In accordance with these structural changes, serum ANP levels (Figure 2O) and the transcript levels of hypertrophy-associated genes (*Myh7*, *Nppa*, and *Nppb*) were significantly decreased in OTUD7B^CKO^ mice following Ang II stimulation (Figure 2P-R). These results establish cardiomyocyte OTUD7B as a critical driver of Ang II-induced cardiac hypertrophy and dysfunction.

### Cardiomyocyte-specific OTUD7B deletion attenuates TAC-induced cardiac hypertrophy

**Figure 3:**
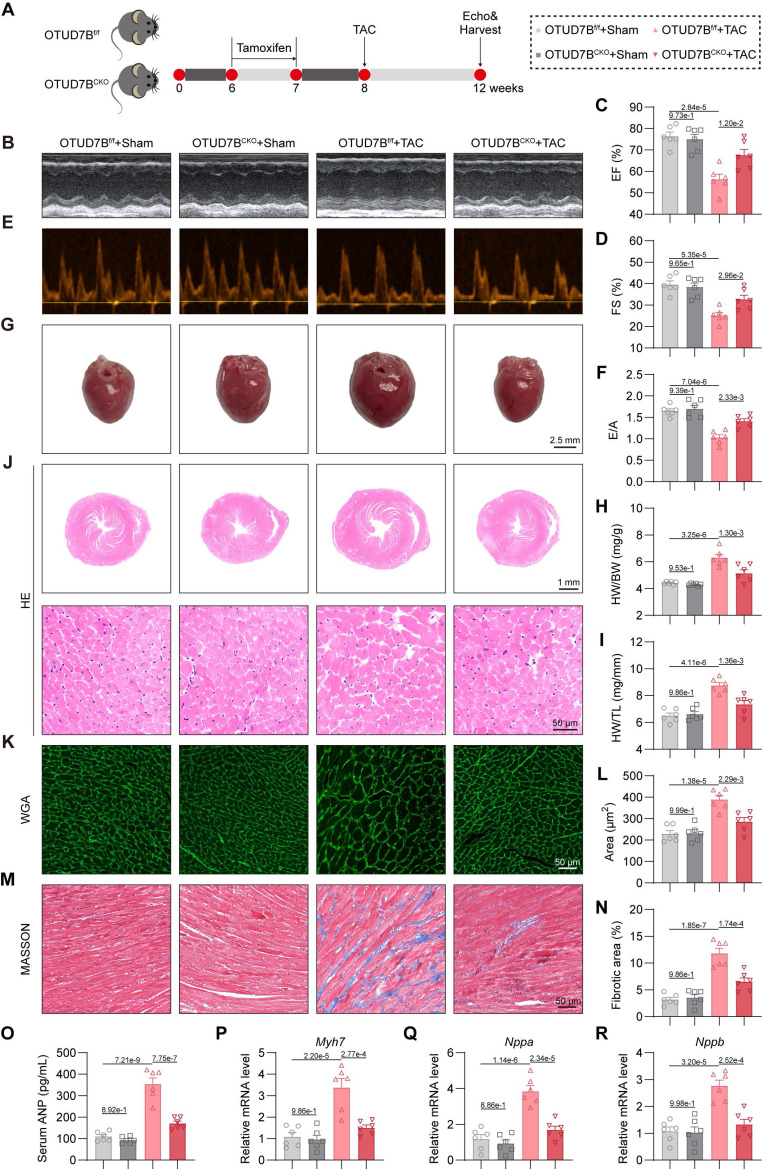
** Cardiomyocyte-specific OTUD7B deletion attenuates TAC-induced cardiac hypertrophy.** (A) Schematic diagram of the TAC-induced mouse model. OTUD7B^f/f^ mice and OTUD7B^CKO^ mice were intraperitoneally injected with tamoxifen for 1 week. Another week later, mice were subjected to TAC. After 4 weeks, the cardiac function of mice was assessed using echocardiography. Mice were then euthanized, and samples were harvested. (B) Representative left ventricular M-mode echocardiographic images. (C-D) Values of ejection fraction (C) and fractional shortening (D). EF, ejection fraction; FS, fractional shortening; n = 6. (E-F) Representative left ventricular PW Doppler echocardiographic images (E) and values of E/A (F). n = 6. (G) Representative whole heart images. (H) The ratio of heart weight to body weight (HW/BW). n = 6. (I) The ratio of heart weight to tibial length (HW/TL). n = 6. (J) Representative HE-stained images of cardiac tissue sections. (K-L) Representative images (K) and quantification (L) from wheat germ agglutinin (WGA)- stained cardiac tissue sections (n = 6). (M-N) Representative images (M) and quantification (N) from Masson's trichrome-stained cardiac tissue sections (n = 6). (O) Serum atrial natriuretic peptide (ANP) levels were detected using ELISA kits (n = 6). (P-R) mRNA levels of *Myh7* (P), *Nppa* (Q), and *Nppb* (R) in heart tissues (n = 6). Signal intensities were adjusted using *Actb* as the normalization control.

We next evaluated the influence of cardiomyocyte OTUD7B deficiency in a pressure overload-mediated cardiac hypertrophy model induced by TAC (Figure 3A). Echocardiography demonstrated preserved cardiac function in OTUD7B^CKO^ mice compared to OTUD7B^f/f^ mice under TAC conditions (Figure 3B-F and Table S5). Morphometric and histological analyses revealed reduced cardiac hypertrophy and interstitial fibrosis in the knockout group (Figure 3G-N). Consistently, serum ANP concentration (Figure 3O) and myocardial expression of hypertrophy-associated genes were markedly decreased in OTUD7B-deficient mice (Figure 3P-R). Together, these results across two myocardial hypertrophy models indicate that cardiomyocyte OTUD7B functions as a pathological regulator of cardiac hypertrophy and dysfunction.

### OTUD7B promotes Ang II-induced cardiomyocyte hypertrophy through its catalytic site C194

**Figure 4:**
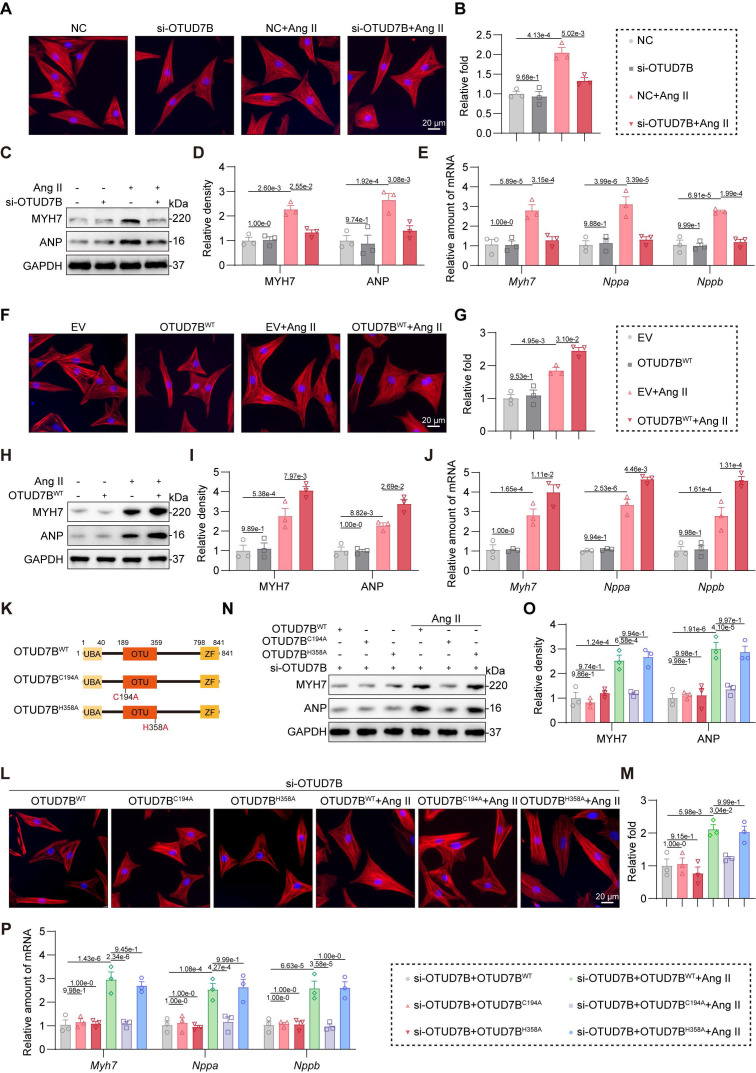
** OTUD7B promotes Ang II-induced cardiomyocyte hypertrophy through its catalytic site C194.** (A-B) TRITC-phalloidin fluorescent staining (A) and quantitative analysis (B) of cell area in NRCMs. NRCMs transfected with OTUD7B siRNA (si-OTUD7B) or negative control (NC) siRNA were stimulated with Ang II for 24 h. (C-D) Western blotting detection (C) and quantitative analysis (D) of MYH7 and ANP. GAPDH served as the internal loading reference (n = 3). NRCMs transfected with si-OTUD7B or NC were challenged with Ang II for 24 h. (E) Transcript levels of *Myh7, Nppa*, and* Nppb* were assessed using RT-qPCR (n = 3). Signal intensities were adjusted using *Actb* as the normalization control. NRCMs transfected with si-OTUD7B or NC were challenged with Ang II for 24 h. (F-G) TRITC-phalloidin fluorescent staining (F) and quantitative analysis (G) of cell area in NRCMs. NRCMs transfected with OTUD7B^WT^ or empty vector (EV) were exposed to Ang II for 24 h. (H-I) Western blotting detection (H) and quantitative analysis (I) of MYH7 and ANP. GAPDH served as the internal loading reference (n = 3). NRCMs expressing OTUD7B^WT^ or EV were exposed to Ang II for 24 h. (J) Transcript levels of *Myh7, Nppa*, and* Nppb* were assessed using RT-qPCR (n = 3). Signal intensities were adjusted using *Actb* as the normalization control. NRCMs transfected with OTUD7B^WT^ or EV were challenged with Ang II for 24 h. (K) Schematic representation for OTUD7B domain architecture together with constructs harboring mutations at the catalytic site. (L-M) TRITC-phalloidin fluorescent staining (L) and quantitative analysis (M) of cell area in NRCMs. NRCMs transfected with si-OTUD7B, OTUD7B^WT^, OTUD7B^C194A^, or OTUD7B^H358A^ were challenged with Ang II for 24 h. (N-O) Western blotting detection (N) and quantitative analysis (O) of MYH7 and ANP. GAPDH served as the internal loading reference (n = 3). NRCMs transfected with si-OTUD7B, OTUD7B^WT^, OTUD7B^C194A^, or OTUD7B^H358A^ were challenged with Ang II for 24 h. (P) Transcript levels of *Myh7, Nppa*, and* Nppb* were assessed using RT-qPCR (n = 3). Signal intensities were adjusted using *Actb* as the normalization control. NRCMs transfected with si-OTUD7B, OTUD7B^WT^, OTUD7B^C194A^ or OTUD7B^H358A^ were challenged with Ang II for 24 h.

To directly investigate the role of OTUD7B in cardiomyocytes, we silenced OTUD7B using specific siRNA in NRCMs (Figure S3A). OTUD7B knockdown suppressed Ang II-induced cell enlargement (Figure 4A-B) and diminished both protein and mRNA expression of hypertrophic markers (Figure 4C-E). Conversely, when we overexpressed OTUD7B in NRCMs using OTUD7B^WT^ plasmid transfection (Figure S3B), OTUD7B overexpression potentiated Ang II-induced cardiomyocyte hypertrophic growth (Figure 4F-G) and gene induction (Figure 4H-J).

Generally, OTUD7B functions as a DUB through its deubiquitinating active site. To determine whether the function of OTUD7B in cardiomyocyte requires enzymatic activity, we constructed two catalytically inactive mutants (OTUD7B^C194A^ and OTUD7B^H358A^) 25 in which cysteine 194 or histidine 358 was replaced by alanine, respectively (Figure 4K). We equally transfected OTUD7B^WT^, OTUD7B^C194A^, and OTUD7B^H358A^ plasmids into NRCMs upon endogenous OTUD7B knockdown (Figure S3C). As shown in Figure 4L-P, inactive mutation of C194 abolished Ang II-induced hypertrophic phenotypes in cardiomyocytes, suggesting that the catalytic site C194 and the deubiquitinase activity of OTUD7B are essential for its pro-hypertrophic function. Collectively, these findings indicate that OTUD7B facilitates Ang II-evoked cardiomyocyte hypertrophy dependent on its catalytic site C194 and DUB activity.

### OTUD7B directly binds to SERCA2a

**Figure 5:**
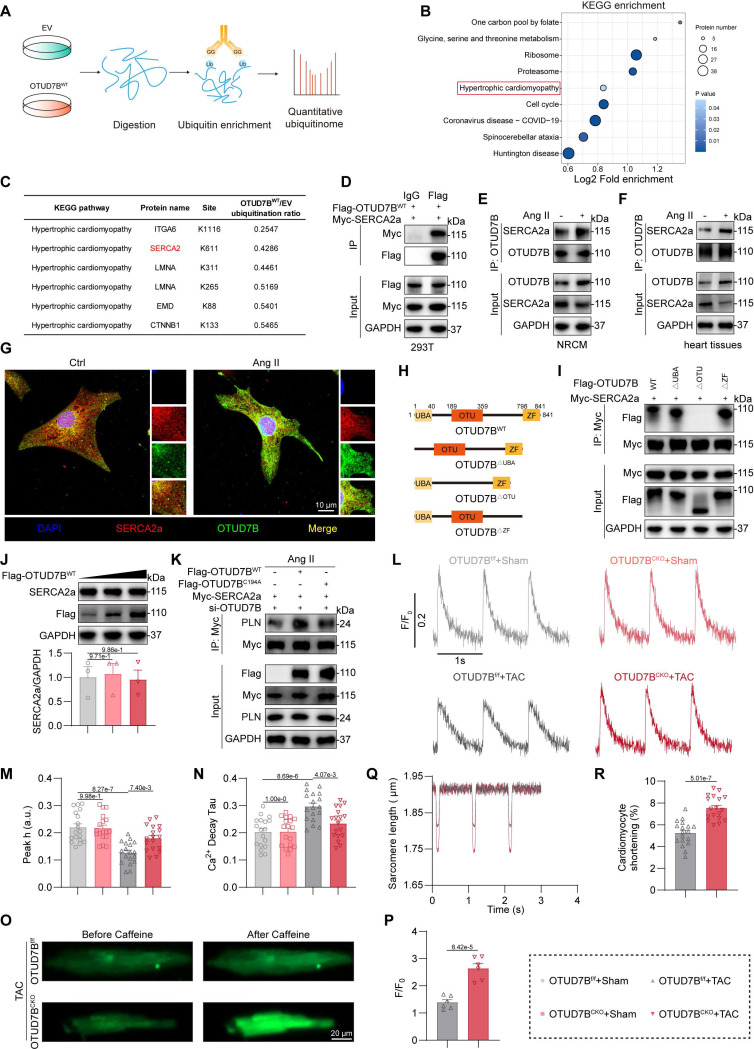
** OTUD7B binds to SERCA2a and regulates Ca^2+^ handling in cardiomyocytes.** (A) Diagrammatic overview of the quantitative ubiquitinome strategy employed to uncover OTUD7B target proteins. (B) KEGG enrichment of proteins with reduced ubiquitination modification in the OTUD7B^WT^ group compared to the EV group. (C) TOP 5 proteins associated with hypertrophic cardiomyopathy and their downregulated ubiquitination lysine residues. (D) Interaction between OTUD7B and SERCA2a was examined in 293T cells co-expressing Flag-OTUD7B^WT^ and Myc-SERCA2a. Protein complexes were isolated using an anti-Flag antibody, with IgG serving as the negative control. IgG, immunoglobulin G. (E) Interaction between OTUD7B and SERCA2a was examined in NRCMs stimulated with Ang II for 24h. OTUD7B was immunoprecipitated by anti-OTUD7B antibody. (F) Co-immunoprecipitation of OTUD7B and SERCA2a in heart tissues of Ang II-treated mice. OTUD7B was immunoprecipitated by anti-OTUD7B antibody. (G) Representative immunofluorescence staining of OTUD7B (green) and SERCA2a (red) in NRCMs challenged with Ang II for 24h. (H) Representation of OTUD7B protein architecture alongside constructs containing targeted domain deletions. (I) Interaction between OTUD7B and SERCA2a was examined in 293T cells co-expressing Myc-SERCA2a, Flag-OTUD7B, and three OTUD7B mutants. Protein complexes were isolated using an anti-Myc antibody. (J) Western blotting detection and quantitative analysis of SERCA2a. 293T cells were transfected with various amounts of Flag-OTUD7B^WT^. GAPDH served as the internal loading reference (n = 3). (K) Interaction between SERCA2a and PLN was examined in NRCMs co-transfected with Myc-SERCA2a, Flag-OTUD7B^WT^, or Flag-OTUD7B^C194A^ and then exposed to Ang II for 24h. Myc-SERCA2a was immunoprecipitated by anti-Myc antibody. (L-N) Representative recordings of Ca^2+^ transients (L), quantification of peak Ca^2+^ amplitude (M), and decay constant (Tau) during the recovery phase of Ca^2+^ transients (N) in isolated adult mouse cardiomyocytes (AMCMs) from OTUD7B^f/f^ and OTUD7B^CKO^ mice under TAC treatment (n = 18). (O-P) Representative images (O) and quantification (P) of calcium release from sarcoplasmic reticulum triggered by caffeine in isolated AMCMs from OTUD7B^f/f^ mice and OTUD7B^CKO^ mice under TAC treatment (n = 6). (Q-R) Sarcomere length change (Q) and quantification (R) of isolated AMCMs from OTUD7B^f/f^ mice and OTUD7B^CKO^ mice under TAC treatment (n = 18).

DUBs exert their biological roles by selectively removing ubiquitin moieties from defined target proteins. To identify molecular substrates of OTUD7B, we conducted quantitative ubiquitinome profiling in NRCMs transfected with empty vector (EV) or OTUD7B^WT^ and challenged with Ang II (Figure 5A). KEGG enrichment analysis of proteins with reduced ubiquitination modification in the OTUD7B^WT^ group compared to EV group highlighted that OTUD7B was associated with the pathway of hypertrophic cardiomyopathy (Figure 5B). The hypertrophic cardiomyopathy pathway was selected due to its direct relevance to the Ang II- or TAC-induced cardiac hypertrophy and dysfunction in this study, while other more statistically significant terms were not related to it. Among the top 5 proteins in the “hypertrophic cardiomyopathy” term (Figure 5C), SERCA2 is a key transporter regulating sarcoplasmic reticulum (SR) Ca^2+^ influx and has three isoforms (SERCA2a, SERCA2b, and SERCA2c), in which only SERCA2a is restricted to cardiomyocytes and has been widely recognized as a key protein in maintaining Ca^2+^ handling in cardiomyocytes and cardiomyocyte function 26. Therefore, we hypothesized that SERCA2a may represent a direct target of OTUD7B.

Co-immunoprecipitation confirmed exogenous and endogenous interaction of OTUD7B with SERCA2a in 293T cells, NRCMs, and mouse heart tissues (Figure 5D-F). Immunofluorescence further revealed colocalization of OTUD7B and SERCA2a in NRCMs after Ang II stimulation (Figure 5G). OTUD7B protein contains a ubiquitin-associated (UBA) domain, an ovarian tumor (OTU) domain, and a zinc finger (ZF) domain 25. To elucidate the precise mechanism of interaction between OTUD7B and SERCA2a, we employed a panel of specifically engineered plasmid constructs (Figure 5H). As shown in Figure 5I, OTUD7B interacted with SERCA2a via its OTU domain.

### OTUD7B promotes SERCA2a-PLN interaction to limit SERCA2a activity in cardiomyocytes

DUBs frequently influence their substrates by modulating either their degradation or functional properties 27. To explore this in the case of OTUD7B, we first assessed whether it could alter the protein abundance of SERCA2a in cells. Our analyses showed that OTUD7B overexpression did not affect the expression or stability levels of SERCA2a. (Figure 5J and Figure S4). In addition, cardiomyocyte-specific deletion of OTUD7B did not alter SERCA2a levels in mouse heart tissues (Figure S5A-C). These findings indicate that OTUD7B does not regulate the stability of SERCA2a in cardiomyocytes. We next investigated whether OTUD7B modulates the activity and function of SERCA2a. Given that SERCA2a activity in cardiomyocytes is controlled by phospholamban (PLN), which binds SERCA2a and decreases its apparent Ca²⁺ affinity 28, 29, we examined the effect of OTUD7B on SERCA2a-PLN interaction in NRCMs. OTUD7B overexpression enhanced SERCA2a-PLN association in NRCMs under Ang II stimulation, whereas the catalytically inactive mutant OTUD7B^C194A^ failed to promote complex formation (Figure 5K). In contrast, cardiomyocyte-specific deletion of OTUD7B resulted in a reduction of SERCA2a-PLN binding in Ang II-treated mouse hearts (Figure S5D). These data demonstrate that OTUD7B associates with SERCA2a and facilitates SERCA2a-PLN interaction, thereby potentially restricting the Ca²⁺-handling function of SERCA2a.

To further evaluate the role of OTUD7B in Ca²⁺ regulation and cardiomyocyte contractility, we isolated adult mouse cardiomyocytes (AMCMs) from OTUD7B^f/f^ and OTUD7B^CKO^ mice subjected to TAC operation. In TAC-challenged AMCMs, OTUD7B deficiency significantly increased Ca²⁺ transient amplitude and shortened the decay constant (Tau), indicating enhanced sarcoplasmic reticulum (SR) Ca²⁺ reuptake (Figure 5L-N). In addition, caffeine-induced Ca²⁺ release was augmented in cardiomyocytes from TAC-operated OTUD7B^CKO^ mice, reflecting an increase in SR Ca²⁺ storage (Figure 5O-P). Similarly, OTUD7B knockdown improved Ca²⁺ release during spontaneous beating in Ang II-treated NRCMs (Figure S6). In line with these changes in Ca²⁺ dynamics, OTUD7B deletion also alleviated contractile dysfunction in TAC-treated cardiomyocytes (Figure 5Q-R). Taken together, these observations establish that OTUD7B impairs SERCA2a activity and Ca²⁺ handling in cardiomyocytes by enhancing SERCA2a-PLN interaction.

### OTUD7B deubiquitinates SERCA2a at K628 to facilitate SERCA2a-PLN interaction

**Figure 6:**
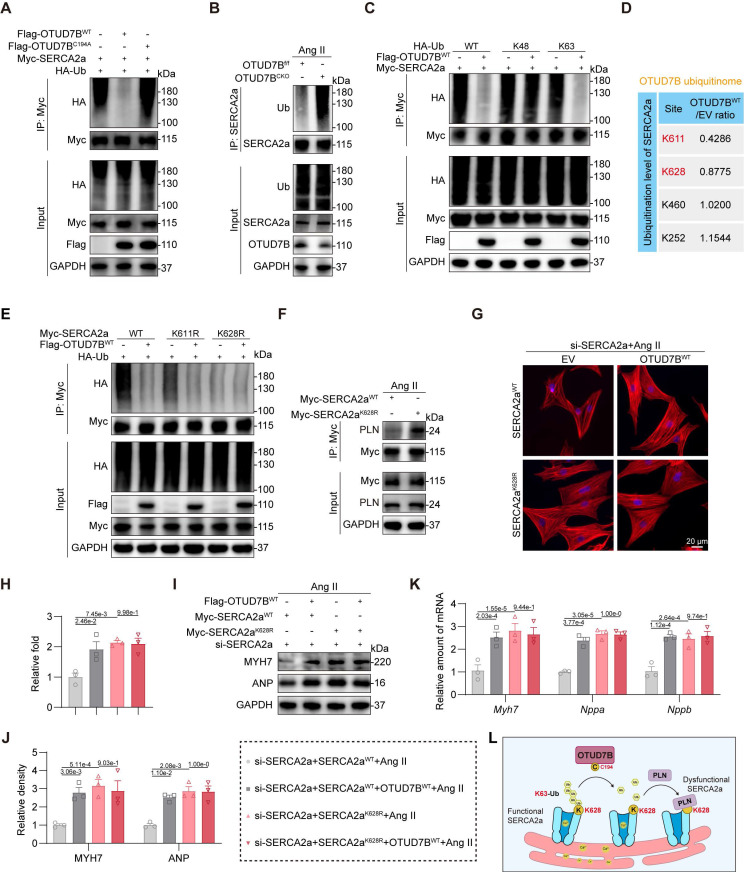
** OTUD7B deubiquitinates SERCA2a at K628 to facilitate SERCA2a-PLN interaction.** (A) Immunoprecipitation of SERCA2a in 293T cells co-transfected with Myc-SERCA2a, HA-Ub, Flag-OTUD7B^WT^, or Flag-OTUD7B^C194A^. Ubiquitinated SERCA2a was detected by immunoblotting using an anti-HA antibody. (B) Immunoprecipitation of SERCA2a in hearts from OTUD7B^f/f^ mice and OTUD7B^CKO^ mice under Ang II treatment. Ubiquitinated SERCA2a was detected by immunoblotting using an anti-Ub antibody. (C) Immunoprecipitation of SERCA2a in 293T cells co-transfected with Myc-SERCA2a, Flag-OTUD7B^WT^, and HA-Ub-WT, HA-Ub-K48 (K48 only), or HA-Ub-K63 (K63 only), respectively. Ubiquitinated SERCA2a was detected by immunoblotting using an anti-HA antibody. (D) Ubiquitination level of SERCA2a at its lysine residues in the OTUD7B^WT^ group compared to the EV group from the ubiquitinome. (E) Immunoprecipitation of SERCA2a in 293T cells co-transfected with Flag-OTUD7B^WT^, HA-Ub, and Myc-SERCA2a-WT, Myc-SERCA2a-K611R, or Myc-SERCA2a-K628R, respectively. Ubiquitinated SERCA2a was detected by immunoblotting using an anti-HA antibody. (F) Interaction between SERCA2a and PLN was examined in NRCMs transfected with Myc-SERCA2a^WT^ or Myc-SERCA2a^ K628R^ and then exposed to Ang II for 24h. Protein complexes were isolated using an anti-Myc antibody. (G-H) TRITC-phalloidin fluorescent staining (G) and quantitative analysis (H) of cell area in NRCMs. NRCMs transfected with si-SERCA2a, OTUD7B^WT^, and SERCA2a^WT^ or SERCA2a^K628R^ were challenged with Ang II for 24 h. (I-J) Western blotting detection (N) and quantitative analysis (O) of MYH7 and ANP. GAPDH served as the internal loading reference (n = 3). NRCMs transfected with si-SERCA2a, OTUD7B^WT^, and SERCA2a^WT^ or SERCA2a^K628R^ were challenged with Ang II for 24 h. (K) Transcript levels of *Myh7, Nppa*, and* Nppb* were assessed using RT-qPCR (n = 3). Signal intensities were adjusted using *Actb* as the normalization control. NRCMs transfected with si-SERCA2a, OTUD7B^WT^, and SERCA2a^WT^ or SERCA2a^K628R^ were challenged with Ang II for 24 h. (L) Conceptual diagram depicting how OTUD7B mediates the removal of ubiquitin chains from SERCA2a.

We previously established that OTUD7B drives cardiomyocyte hypertrophy through its deubiquitinating activity. Based on this, we hypothesized that OTUD7B enhances the SERCA2a-PLN interaction by removing ubiquitin modifications from SERCA2a. Indeed, we found that wild-type OTUD7B effectively deubiquitinated SERCA2a, whereas the catalytically inactive mutant failed to eliminate ubiquitin chains (Figure 6A). Consistently, cardiomyocyte-specific deletion of OTUD7B in Ang II-treated mice resulted in elevated SERCA2a ubiquitination in heart tissues (Figure 6B). In order to determine the specific ubiquitin linkage involved, HA-tagged ubiquitin was replaced with mutant variants restricted to either K48 or K63, representing the two predominant canonical ubiquitin modifications. Our results revealed that OTUD7B specifically removed K63-linked, but not K48-linked, ubiquitination from SERCA2a (Figure 6C), demonstrating that OTUD7B mediates K63-dependent deubiquitination of SERCA2a.

We next sought to identify the lysine residue on SERCA2a targeted by OTUD7B. Quantitative ubiquitinome analysis revealed two potential sites, K611 and K628 (Figure 6D and Figure S7). Mutagenesis experiments, in which lysine was substituted with arginine, showed that the K628R mutation reduced overall ubiquitination of SERCA2a, and this reduction was not further affected by OTUD7B (Figure 6E). These findings establish K628 as the critical site for OTUD7B-mediated deubiquitination. Furthermore, the K628R mutation significantly enhanced the SERCA2a-PLN interaction (Figure 6F). We next assessed the functional relevance of SERCA2a K628 deubiquitination. SERCA2a^WT^ and SERCA2a^K628R^ were equally transfected into NRCMs after endogenous SERCA2a knockdown (Figure S8). Both OTUD7B overexpression and the K628R mutation exacerbated Ang II-induced cardiomyocyte enlargement (Figure 6G-H). Importantly, the pro-hypertrophic effect of OTUD7B was abolished in cardiomyocytes expressing the K628R mutant, highlighting that OTUD7B's function requires deubiquitination at K628. These results were corroborated by analysis of hypertrophic marker gene expression at both mRNA and protein levels (Figure 6I-K). Additionally, either OTUD7B overexpression or SERCA2a-K628R mutation suppressed Ca²⁺ release in Ang II-challenged NRCMs during spontaneous contractions, whereas the inhibitory effect of OTUD7B was absent in cells expressing the K628R mutant (Figure S9). Taken together, these findings demonstrate that OTUD7B removes K63-linked ubiquitin chains from SERCA2a at residue K628, thereby facilitating its interaction with PLN. This enhanced SERCA2a-PLN binding constrains SERCA2a activity, ultimately promoting cardiomyocyte hypertrophy (Figure 6L).

### Cardiomyocyte-specific OTUD7B overexpression promotes cardiac hypertrophy by deubiquitinating SERCA2a at K628

**Figure 7:**
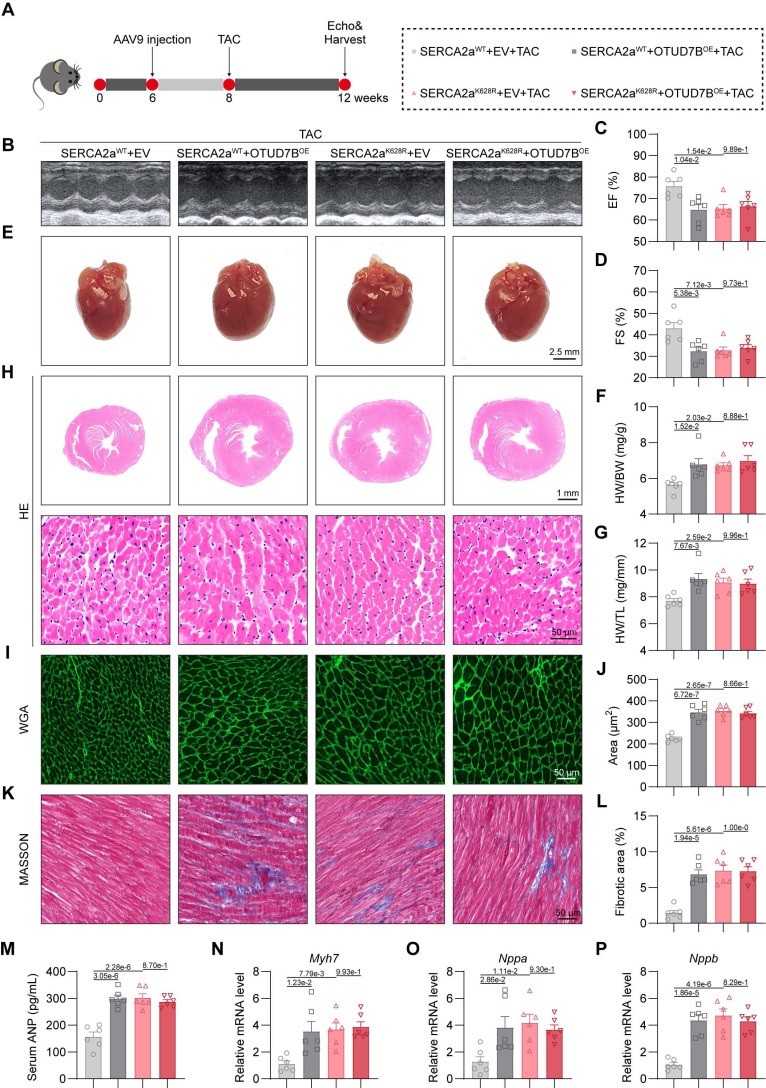
** Cardiomyocyte-specific OTUD7B overexpression promotes cardiac hypertrophy by deubiquitinating SERCA2a at K628.** (A) Schematic diagram of the TAC-induced mouse model. WT mice were injected with AAV9 cardiomyocyte-specific overexpressing SERCA2a^WT^ or SERCA2a^K628R^, and OTUD7B^OE^ or empty vector (EV) by the tail vein. After 2 weeks, mice were subjected to TAC. After 4 weeks, the cardiac function of mice was assessed using echocardiography. Mice were then euthanized, and samples were harvested. (B) Representative left ventricular M-mode echocardiographic images. (C-D) Values of ejection fraction (C) and fractional shortening (D). EF, ejection fraction; FS, fractional shortening; n = 6. (E) Representative whole heart images. (F) The ratio of heart weight to body weight (HW/BW). n = 6. (G) The ratio of heart weight to tibial length (HW/TL). n = 6. (H) Representative HE-stained images of cardiac tissue sections. (I-J) Representative images (I) and quantification (J) of wheat germ agglutinin (WGA)-stained cardiac tissue sections (n = 6). (K-L) Representative images (K) and quantification (L) from Masson's trichrome-stained cardiac tissue sections (n = 6). (M) Serum atrial natriuretic peptide (ANP) levels were detected using ELISA kits (n = 6). (N-P) mRNA levels of *Myh7* (N), *Nppa* (O), and *Nppb* (P) in heart tissues (n = 6). Signal intensities were adjusted using *Actb* as the normalization control.

To evaluate whether OTUD7B promotes cardiac hypertrophy *in vivo* through deubiquitination of SERCA2a at K628, we generated cardiomyocyte-specific overexpression models for OTUD7B (OTUD7B^OE^) and SERCA2a (SERCA2a^WT^ or SERCA2a^K628R^) using adeno-associated virus serotype 9 (AAV9) vectors driven by the cardiac-specific cTNT promoter. These AAV9 constructs were delivered to mice via tail-vein injection, followed two weeks later by transverse aortic constriction (TAC) surgery for an additional four weeks (Figure 7A). Previous studies have shown that SERCA2a overexpression alleviates cardiac hypertrophy and contractile impairment 11, 30. We confirmed robust upregulation of OTUD7B, SERCA2a^WT^, or SERCA2a^K628R^ proteins in myocardial tissues, and noted that OTUD7B overexpression did not alter the SERCA2a abundance *in vivo* (Figure S10).

Echocardiographic analysis demonstrated that either OTUD7B or SERCA2a^K628R^ overexpression exacerbated TAC-induced cardiac dysfunction. However, concurrent OTUD7B overexpression in SERCA2a^K628R^-overexpressing mice did not result in further deterioration in cardiac performance (Figure 7B-D and Table S6). Both OTUD7B and SERCA2a^K628R^ overexpression resulted in an enlarged gross heart size and increased ratios of heart weight to body weight and to tibial length, yet these hypertrophic effects were not further intensified when both constructs were co-expressed (Figure 7E-G). Histopathological examinations revealed marked cardiomyocyte hypertrophy (Figure 7H-J) and enhanced interstitial fibrosis (Figure 7K-L) in hearts overexpressing OTUD7B or SERCA2a^K628R^, but no additive effect was observed upon dual expression. Similar results were reflected in serum ANP concentrations (Figure 7M) and in the transcriptional upregulation of hypertrophic markers (Figure 7N-P). Collectively, these findings demonstrate that cardiomyocyte OTUD7B induces cardiac hypertrophy and functional impairment through deubiquitination of SERCA2a at K628.

## Discussion

In the present work, we found that OTUD7B levels were upregulated in cardiomyocytes from hypertrophic hearts of both mice and humans. Targeted deletion of OTUD7B in cardiomyocytes attenuated cardiac hypertrophy and functional impairment induced by Ang II or TAC. Comprehensive quantitative ubiquitinome profiling revealed SERCA2a as a principal substrate of OTUD7B. OTUD7B removed K63-linked ubiquitin chains from SERCA2a at K628 through its catalytic site C194, thereby strengthening SERCA2a-PLN interaction and impairing Ca²⁺ handling. Moreover, cardiomyocyte-specific OTUD7B overexpression aggravated TAC-induced cardiac hypertrophy by targeting SERCA2a at K628.

Most mammalian cardiomyocytes are terminally differentiated, making preservation of protein function central to cardiac health 31. Among the regulatory mechanisms, DUB-mediated deubiquitination plays a central role in controlling protein stability and activity, thereby shaping cardiovascular homeostasis 27. Several DUBs have been implicated in cardiac hypertrophy-for instance, ubiquitin-specific protease 10 (USP10) exerts protective effects by reducing SIRT6 abundance in pressure-overloaded hearts 32, and ubiquitin thioesterase OTU1 (YOD1) promoted cardiac hypertrophy by deubiquitinating STAT3 33. Despite the large number of DUBs encoded in the human genome, only a minority have been functionally investigated in cardiac hypertrophy, underscoring the need to uncover additional candidates. Here, we identified that cardiomyocyte-derived OTUD7B promotes pathological cardiac hypertrophy and dysfunction. The absence of an overt phenotype in cardiomyocyte-specific OTUD7B deficient mice under sham conditions suggests that OTUD7B is not required for maintaining basal cardiac structure and function. Instead, our data demonstrate that OTUD7B acts as a stress-responsive regulator that becomes functionally relevant under pathological conditions. Consistently, we observed that OTUD7B expression was markedly upregulated in hypertrophic hearts following TAC, whereas its expression remains low under sham conditions. However, the mechanism by which hypertrophic stimuli induce OTUD7B expression in cardiomyocytes is unclear. We observed that *Otud7b* mRNA was upregulated in hypertrophic hearts from both mice and humans, indicating that OTUD7B may be regulated at the transcriptional level by a certain transcription factor during pathological cardiac hypertrophy. In addition, pathological hypertrophy is associated with epigenetic remodeling, including changes in chromatin accessibility and histone modifications 34, which may further facilitate OTUD7B upregulation in hypertrophic myocardium. Elucidating the signaling and transcriptional mechanisms governing OTUD7B induction will be an important direction for future investigation.

OTUD7B belongs to the OTU family of DUBs and is originally characterized as a critical regulator of immune signalling through deubiquitination of TRAF3, thereby restraining non-canonical NF-κB activation 12. Beyond this canonical role, OTUD7B has been implicated in oncogenic programs by stabilizing key substrates. For instance, OTUD7B has been shown to promote breast cancer progression through stabilizing estrogen receptor-α (ERα) 35, and to facilitate mTOR complex 2 (mTORC2) activation by removing K63-linked ubiquitin from the GβL subunit 36. Our results provide strong evidence that cardiomyocyte-derived OTUD7B drives cardiac hypertrophy and dysfunction. Importantly, the deubiquitinating activity of OTUD7B, governed by its catalytic site C194, is essential for its pro-hypertrophic function. This suggests that pharmacological inhibition of OTUD7B activity may represent a viable therapeutic approach against cardiac hypertrophy. Mechanistically, we demonstrated that OTUD7B removes K63-linked ubiquitin from SERCA2a, thereby promoting SERCA2a-PLN complex formation and suppressing Ca²⁺ handling in cardiomyocytes. These insights expand the known biological roles of OTUD7B and establish it as a novel pathogenic amplifier of cardiac hypertrophy.

Notably, two recent studies reported that OTUD7B alleviates TAC-induced cardiac hypertrophy 37, 38, which contrasted with our findings showing that cardiomyocyte-derived OTUD7B exacerbates pathological cardiac hypertrophy and dysfunction. This discrepancy is most likely due to differences in the genetic strategies of mice and experimental cell models. The previous two studies used systemic modulation of OTUD7B (global OTUD7B knockout mice and AAV9-induced OTUD7B knockdown mice, respectively), where non-cardiomyocyte cell types may influence cardiac outcomes. Our scRNA-seq analysis revealed that OTUD7B was primarily expressed in cardiomyocytes, but was also expressed to a lesser extent in fibroblasts and endothelial cells. Therefore, the results of global OTUD7B knockout or knockdown mice could be affected by the actions of fibroblasts and endothelial cells. In contrast, our work employed cardiomyocyte-specific OTUD7B deletion and overexpression manipulation, revealing a direct pro-hypertrophic effect of cardiomyocyte-derived OTUD7B. Moreover, we utilized two models (Ang II and TAC) of myocardial hypertrophy to validate our findings. In the cell experiments, the previous two studies used phenylephrine (PE), an α1-adrenergic receptor agonist that predominantly activates adrenergic signaling and triggers rapid, transient hypertrophic responses 39, while our work used Ang II, a vasoactive peptide that mainly engages AT1R-mediated signaling pathways and induces sustained hypertrophy 40. These distinct upstream mechanisms may lead to different downstream targets of OTUD7B, resulting in opposite outcomes. Collectively, these differences suggest that OTUD7B may exert context-dependent effects in cardiac hypertrophy, emphasizing the need for careful dissection of cell type- and stimulus-specific mechanisms of OTUD7B in cardiac hypertrophy in future studies.

SERCA2a, a cardiac-specific isoform of the sarcoplasmic/endoplasmic reticulum Ca²⁺-ATPase, is fundamental for maintaining intracellular calcium balance and ensuring effective excitation-contraction coupling 28, 41. Proper SERCA2a activity is essential for Ca²⁺ reuptake into the sarcoplasmic reticulum during diastole, thereby preserving contractility 42. PLN serves as a negative regulator by lowering SERCA2a's Ca²⁺ affinity 28. Impaired SERCA2a activity and dysregulated Ca²⁺ cycling are hallmark features of cardiac hypertrophy and failure 43. Our data showed that OTUD7B-mediated K63 deubiquitination of SERCA2a strengthens SERCA2a-PLN binding without changing SERCA2a abundance, impairing Ca²⁺ cycling and driving cardiac hypertrophy. The post-translational regulation of SERCA2a has gained increasing attention in recent years. K460 and K541 ubiquitination promotes degradation 44; hydrogen sulfide 45 and succinylation 46 pathways also modulate its stability. Our group previously found USP25 11 and JOSD2 47 protect SERCA2a stability by K48-specific deubiquitination. The present work identifies K628 as a critical K63-linked ubiquitination site removed by OTUD7B. The K628R mutation abolished this ubiquitination and altered SERCA2a's Ca²⁺-handling ability, thereby producing hypertrophic effects. We proposed that OTUD7B-mediated deubiquitination may expose the K628 region, enhancing PLN binding and promoting SERCA2a inhibition. This mode of regulation reveals a novel mechanism distinct from proteolytic regulation of SERCA2a and PLN phosphorylation-based control of the SERCA2a-PLN complex 48.

From a therapeutic perspective, these findings identify the OTUD7B-SERCA2a-PLN axis as a novel therapeutic target for cardiac hypertrophy. Although SERCA2a overexpression has shown cardioprotective effects in preclinical models, the translation of this to clinical therapy has faced challenges due to insufficient gene transfer efficacy 41, 49, 50. Unlike conventional strategies aiming to increase SERCA2a abundance, inhibiting OTUD7B-via genetic ablation or small molecules targeting its catalytic site C194-could enhance SERCA2a function more subtly through endogenous modulation. Given OTUD7B's low basal expression but marked upregulation in cardiomyocytes from hypertrophic hearts, investigating selective OTUD7B inhibitors and targeted delivery of such inhibitors may achieve therapeutic benefits with minimal side effects.

In summary, this study reveals that cardiomyocyte-derived OTUD7B promotes cardiac hypertrophy via K63-linked deubiquitination of SERCA2a at K628, reinforcing SERCA2a-PLN interaction and disturbing Ca²⁺ homeostasis. These findings establish OTUD7B as a novel pathological regulator and promising therapeutic target of cardiac hypertrophy and dysfunction.

## Supplementary Material

Supplementary figures and tables.

## Abbreviations

AAV9: adeno-associated virus 9
AMCMs: adult mouse cardiomyocytes
Ang II: angiotensin II
ANP: atrial natriuretic peptide
DUBs: deubiquitinating enzymes
EF: ejection fraction
EV: empty vector
FS: fractional shortening
HE: hematoxylin and eosin
MPMs: mouse primary peritoneal macrophages
MYH7: myosin heavy chain 7
NC: negative control
NRCFs: neonatal rat cardiac fibroblasts
NRCMs: neonatal rat cardiomyocytes
OTUD7B: ovarian tumor domain-containing 7B
PLN: phospholamban
SERCA2a: sarcoplasmic/endoplasmic reticulum Ca^2+^ ATPase 2a
TAC: transverse aortic constriction
WGA: Wheat germ agglutinin
WT: wild type
