# Supplementary material for: Cardiomyocyte-derived OTUD7B promotes cardiac hypertrophy by deubiquitinating SERCA2a
Source: Theranostics. 2026 Feb 4;16(8):4452–70. doi: 10.7150/thno.129105 (PMC12906209; doi:10.7150/thno.129105)
Supplement: Supplementary file 1 — Supplementary figures and tables. [file thnov16p4452s1.pdf]

Supplemental Material

Cardiomyocyte-derived OTUD7B promotes cardiac hypertrophy by deubiquitinating SERCA2a

The Supplemental Material includes 10 figures and 6 tables.

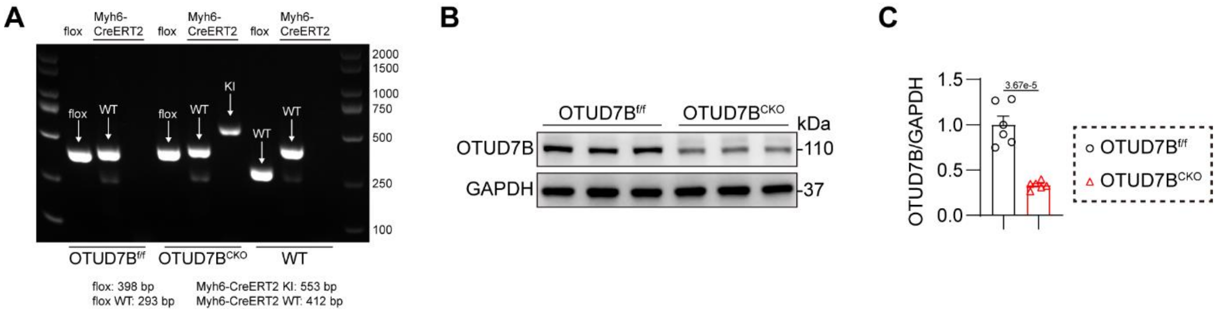

Supplementary Figure S1. Generation of cardiomyocyte-specific OTUD7B knockout mice ($OTUD7B^{CKO}$). (A) To identify the genotypes of $OTUD7B^{f/f}$ and $OTUD7B^{CKO}$ mice, cDNA was extracted from mouse tail and the primers for identification of $OTUD7B^{f/f}$ and $OTUD7B^{CKO}$ mice were used for PCR assay. (B-C) Western blotting detection (B) and quantitative analysis (C) of OTUD7B in heart tissues of $OTUD7B^{f/f}$ mice and $OTUD7B^{CKO}$ mice. GAPDH served as the internal loading reference (n = 6). $OTUD7B^{f/f}$ mice and $OTUD7B^{CKO}$ mice were intraperitoneally injected with tamoxifen for 1 week. Another week later, mice were euthanized and samples were harvested.

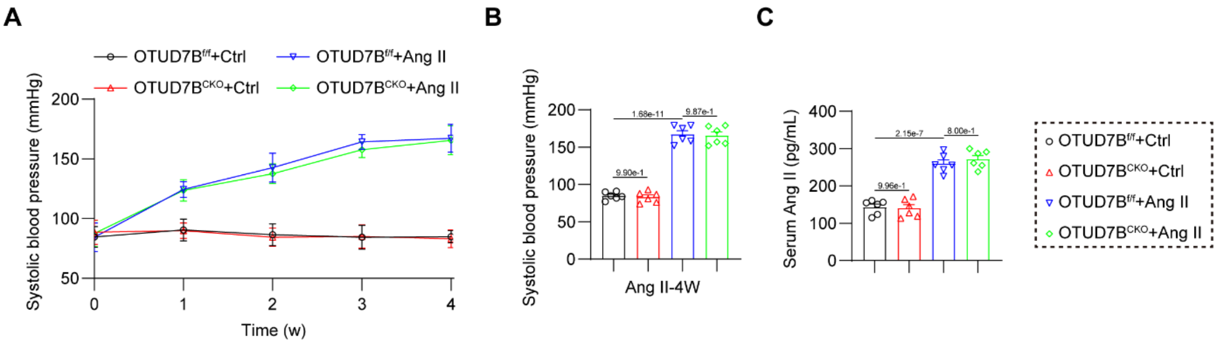

Supplementary Figure S2. Cardiomyocyte-specific OTUD7B deletion did not affect systolic blood pressure and Ang II level in Ang II-induced mice. (A) Systolic blood pressure was recorded during the Ang II-challenged mouse experiments (n = 6). (B) Systolic blood pressure of mice in the fourth week of Ang II treatment (n = 6). (C) Serum Ang II level was analyzed using ELISA (n = 6).

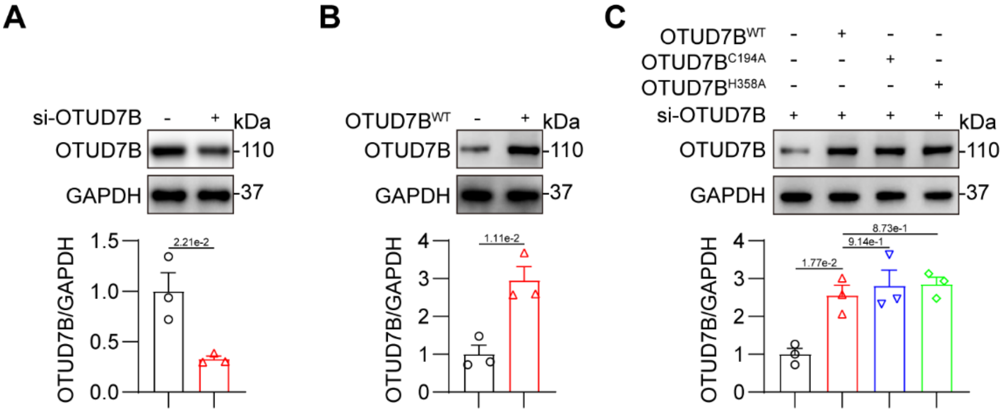

Supplementary Figure S3. Effects of OTUD7B knockdown or overexpression in NRCMs. (A) NRCMs were exposed to OTUD7B-targeting siRNA (si-OTUD7B) for 24 hours, while control cells received a non-targeting siRNA (NC). OTUD7B protein expression was assessed by Western blotting (n = 3). (B) NRCMs were exposed to OTUD7B^{WT} or empty vector (EV) for 24h. OTUD7B protein expression was assessed by Western blotting (n = 3). (C) NRCMs were exposed to si-OTUD7B, OTUD7B^{WT}, OTUD7B^{C194A} or OTUD7B^{H358A} for 24h. OTUD7B protein expression was assessed by Western blotting (n = 3).

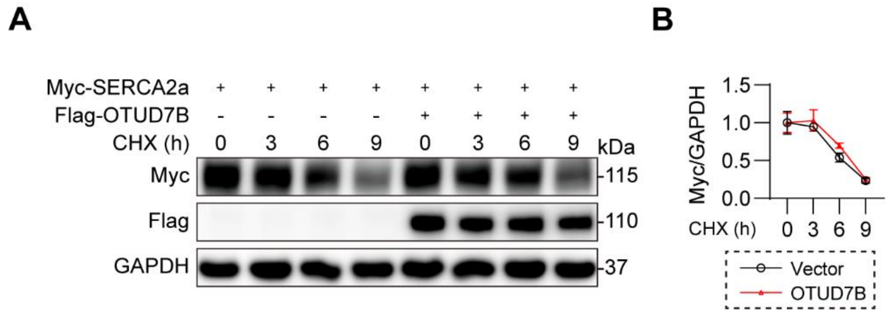

Supplementary Figure S4. OTUD7B does not affect the protein stability of SERCA2a. (A-B) Western blotting detection (A) and quantitative analysis (B) of Myc. 293T cells transfected with Flag-OTUD7B and Myc-SERCA2a were treated with cycloheximide (CHX, 50ug/ml) at the indicated time points. GAPDH was used as the loading control (n = 3).

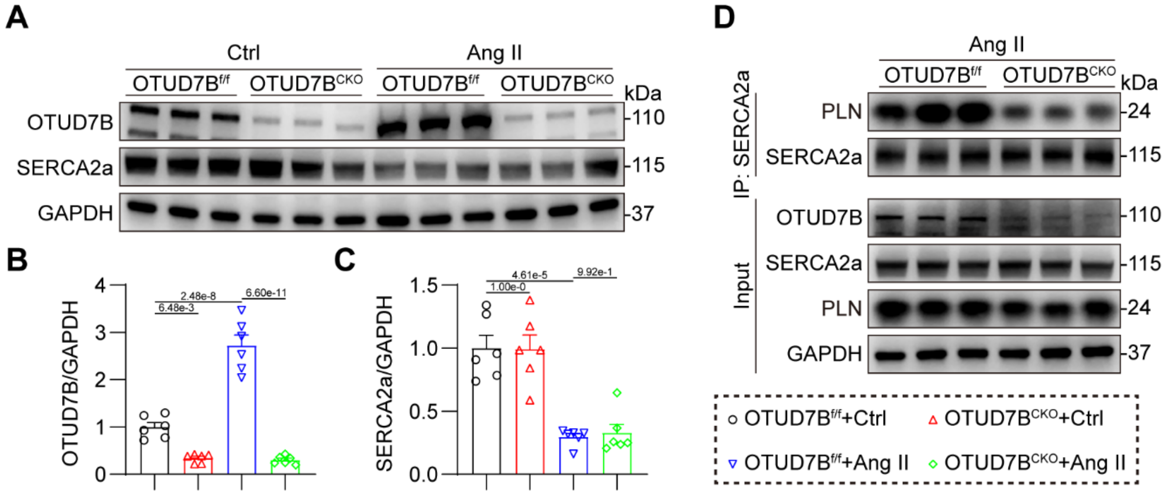

Supplementary Figure S5. Cardiomyocyte-specific OTUD7B deletion inhibits the binding of SERCA2a and PLN in Ang II-induced cardiac hypertrophy. (A-C) Western blotting detection (A) and quantitative analysis (B-C) of OTUD7B and SERCA2a in heart tissues (n = 6). (D) Co-immunoprecipitation of SERCA2a and PLN in heart tissues. SERCA2a was immunoprecipitated by anti-SERCA2a antibody.

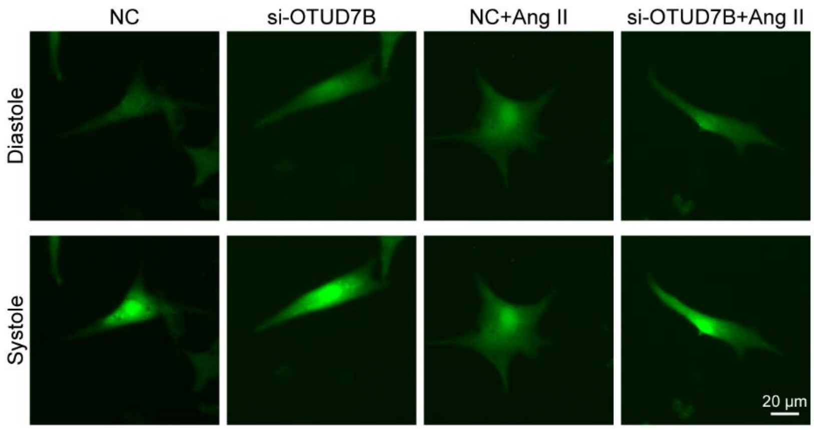

Supplementary Figure S6. OTUD7B regulates sarcoplasmic reticulum (SR) calcium content in cardiomyocytes. Representative confocal images of cytosolic Ca^{2+} determined by the Fluo-4 AM fluorescence signal in NRCMs transfected with si-OTUD7B or NC and then challenged with Ang II for 24h.

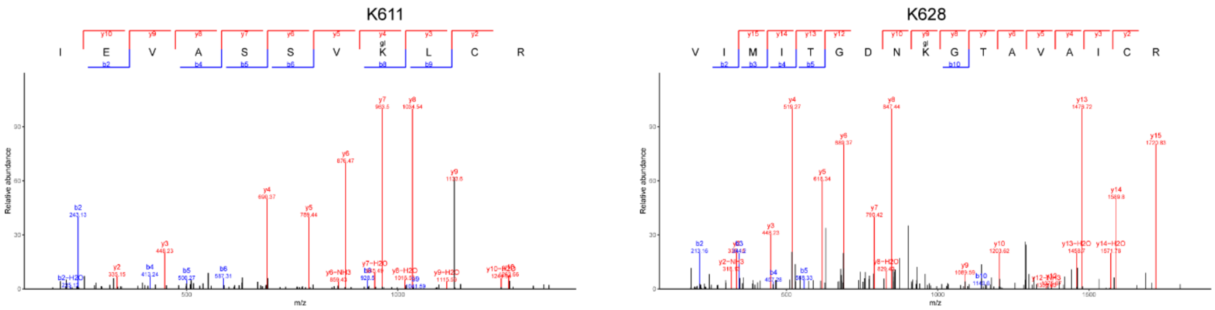

Supplementary Figure S7. Second-order spectrums of the peptide of SERCA2a at K611 and K628.

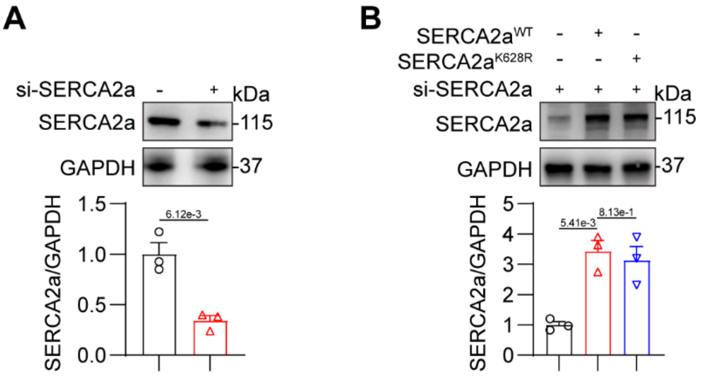

Supplementary Figure S8. Effects of SERCA2a knockdown or overexpression in NRCMs. (A) NRCMs were exposed to SERCA2a siRNA (si-SERCA2a) for 24h, while control cells received NC siRNA. SERCA2a protein expression was assessed by Western blotting (n = 3). (B) NRCMs were exposed to si-SERCA2a, SERCA2a^{WT} or Myc-SERCA2a ^{K628R} for 24h. SERCA2a protein expression was assessed by Western blotting (n = 3).

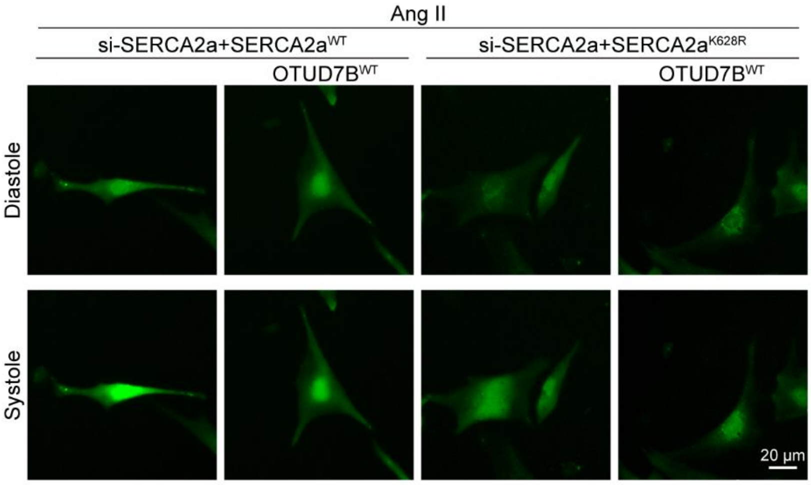

Supplementary Figure S9. OTUD7B regulates sarcoplasmic reticulum (SR) calcium content in cardiomyocytes by regulating SERCA2a deubiquitination at residue K628. Representative confocal images of cytosolic Ca^{2+} determined by the Fluo-4 AM fluorescence signal in NRCMs transfected with si-SERCA2a, $OTUD7B^{WT}$, and $SERCA2a^{WT}$ or $SERCA2a^{K628R}$ and then challenged with Ang II for 24 h.

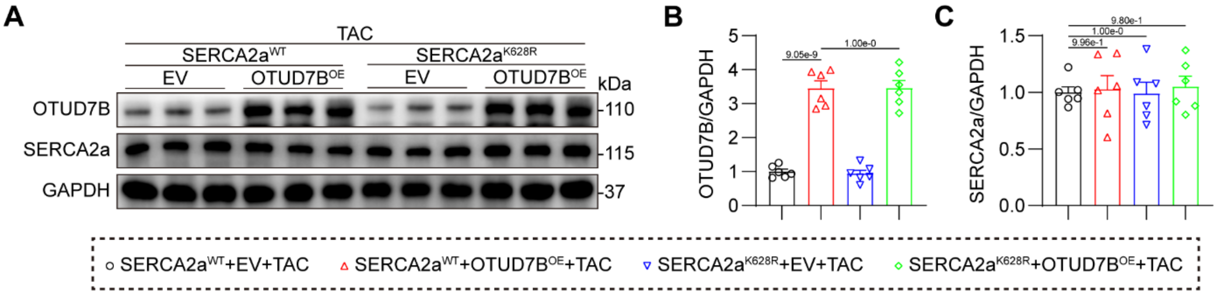

Supplementary Figure S10. Effects of OTUD7B and SERCA2a overexpression in vivo. (A-C) Western blotting detection (A) and quantitative analysis (B-C) of OTUD7B and SERCA2a in heart tissues (n = 6).

Supplementary Table S1. Clinical measurements for the human specimens

Type	Age (years)	Sex	IVS,mm	EF%
NCH-1	66	Male	11	68
NCH-2	48	Male	10	71
NCH-3	53	Female	9	64
NCH-4	81	Male	9	60
NCH-5	52	Male	10	67
NCH-6	76	Male	10	64
CH-1	46	Male	15	50
CH-2	77	Female	16	40
CH-3	73	Female	15	42
CH-4	80	Male	18	32
CH-5	57	Male	17	42
CH-6	55	Male	16	49

NCH, non-cardiac hypertrophy; CH, cardiac hypertrophy; IVS, interventricular septal thickness; EF, ejection fraction.

Supplementary Table S2. Primer sequences for PCR genotyping analysis

Gene	Species	Sequence
Otud7b-flox	Mouse	GTAGCTCACAGTTTCAGCCTGGATATT
		CCCAGCCTGTATGACTCTTTATATCTTC
Myh6-CreERT2 WT	Mouse	CAGCAAAACCTGGCTGTGGATC
		ATGAGCCACCATGTGGGTGTC
Myh6-CreERT2 KI	Mouse	GGCACATGAGTAACAAAGGCATG
		AGCCAACCTTTGTTCATGGCAG

Supplementary Table S3. Primer sequences for real-time qPCR assay

**Gene**	**Species**	**Sequence**
Myh7	Mouse	ACTGTCAACACTAAGAGGGTCA<br>TTGGATGATTTGATCTTCCAGGG
Nppa	Mouse	GCTTCCAGGCCATATTGGAG<br>GGGGGCATGACCTCATCTT
Nppb	Mouse	GAGGTCACTCCTATCCTCTGG<br>GCCATTTCCTCCGACTTTTCTC
Actb	Mouse	GGCTGTATTCCCCTCCATCG<br>CCAGTTGGTAACAATGCCATGT
Otud7b	Mouse	TGCTGTCCTGTCGGATTTTGT<br>TGGACTTGACGCAACTGTTCA
Myh7	Rat	CTGAGGAGACACAGCGTTCT<br>GGGTCAGCTGAGAGATAAGA
Nppa	Rat	GAGCGAGCAGACCGATGAAGC<br>TCCATCTCTCTGAGACGGGTTGAC
Nppb	Rat	AGTCTCCAGAACAATCCACGATGC<br>GCCTTGGTCCTTTGAGAGCTGTC
Actb	Rat	ACTATCGGCAATGAGCGGTTCC<br>TGGCATAGAGGTCTTTACGGATGTC
Myh7	Human	GGAGTTCACACGCCTCAAAGAG<br>TCCTCAGCATCTGCCAGGTTGT
Nppa	Human	CACCGTGAGCTTCCTCCTTT<br>CCAAATGGTCCAGCAAATTCTTG
Actb	Human	CATGTACGTTGCTATCCAGGC<br>CTCCTTAATGTCACGCACGAT
Otud7b	Human	GTCAGATTTTGTCCGTTCCACA<br>CATGGACTTGACGTAGCTGTT

Supplementary Table S4. Echocardiographic data in all groups

	Ctrl		Ang II	
	OTUD7B$^{f/f}$	OTUD7B^{CKO}	OTUD7B$^{f/f}$	OTUD7B^{CKO}
EF (%)	72.96±0.66	71.28±1.39^{ns}	58.6±1.27*	65.83±1.5$^{\#}$
FS (%)	39.34±0.68	37.81±1.38^{ns}	27.64±0.84*	33.12±1.31$^{\#}$
IVSd (mm)	0.81±0.04	0.83±0.04^{ns}	1.08±0.06*	0.88±0.03$^{\#}$
LVIDd (mm)	3.49±0.06	3.49±0.1^{ns}	4.03±0.2^{ns}	3.64±0.2^{NS}
LVPWd (mm)	0.93±0.06	1.0±0.06^{ns}	1.0±0.16^{ns}	1.03±0.07^{NS}
E/A	1.82±0.06	1.74±0.06^{ns}	1.22±0.04*	1.5±0.06$^{\#}$
HR (bpm)	482±7.24	479.5±6.2^{ns}	473.5±5.82^{ns}	484.3±3.02^{NS}
HW/BW (mg/g)	4.49±0.08	4.51±0.06^{ns}	6.26±0.39*	5.15±0.2$^{\#}$
HW/TL (mg/mm)	7.04±0.14	6.74±0.21^{ns}	8.67±0.38*	7.36±0.25$^{\#}$

EF, ejection fraction; FS, fractional shortening; IVSd, diastole interventricular septum; LVIDd, diastole left ventricular internal diameter; LVPWd, diastole left ventricular posterior wall; E/A, E peak/A peak; HR, heart rate; HW, heart weight; BW, body weight; TL, tibial length. Data were shown as mean ± SEM. ns, $p>0.05$ vs OTUD7B$^{f/f}$ + Ctrl; *, $p<0.05$ vs OTUD7B$^{f/f}$ + Ctrl. NS, $p>0.05$ vs OTUD7B$^{f/f}$ + Ang II; #, $p<0.05$ vs OTUD7B$^{f/f}$ + Ang II.

Supplementary Table S5. Echocardiographic data in all groups

	Sham		TAC	
	OTUD7B$^{f/f}$	OTUD7B^{CKO}	OTUD7B$^{f/f}$	OTUD7B^{CKO}
EF (%)	76.4±1.94	75.01±2.21^{ns}	56.32±2.36*	67.62±2.66$^{\#}$
FS (%)	39.59±1.7	38.44±1.85^{ns}	25.22±1.39*	32.69±1.94$^{\#}$
IVSd (mm)	0.77±0.02	0.76±0.04^{ns}	1.02±0.04*	0.78±0.04$^{\#}$
LVIDd (mm)	3.93±0.06	4.01±0.09^{ns}	4.25±0.1^{ns}	4.1±0.14^{NS}
LVPWd (mm)	0.85±0.06	0.9±0.08^{ns}	1.0±0.11^{ns}	0.96±0.07^{NS}
E/A	1.65±0.05	1.7±0.08^{ns}	1.03±0.06*	1.41±0.06$^{\#}$
HR (bpm)	468.5±6.01	477±4.31^{ns}	485.8±10.41^{ns}	479.5±5.7^{NS}
HW/BW (mg/g)	4.43±0.05	4.3±0.04^{ns}	6.3±0.25*	5.15±0.26$^{\#}$
HW/TL (mg/mm)	6.5±0.2	6.61±0.19^{ns}	8.75±0.21*	7.34±0.29$^{\#}$

EF, ejection fraction; FS, fractional shortening; IVSd, diastole interventricular septum; LVIDd, diastole left ventricular internal diameter; LVPWd, diastole left ventricular posterior wall; E/A, E peak/A peak; HR, heart rate; HW, heart weight; BW, body weight; TL, tibial length; TAC, transverse aortic constriction. Data were shown as mean ± SEM. ns, $p>0.05$ vs OTUD7B$^{f/f}$ + Sham; *, $p<0.05$ vs OTUD7B$^{f/f}$ + Sham. NS, $p>0.05$ vs OTUD7B$^{f/f}$ + TAC; #, $p<0.05$ vs OTUD7B$^{f/f}$ + TAC.

Supplementary Table S6. Echocardiographic data in all groups

	TAC			
	SERCA2a^{WT}+ EV	SERCA2a^{WT}+ OTUD7B^{OE}	SERCA2a^{K628R}+ EV	SERCA2a^{K628R}+ OTUD7B^{OE}
EF (%)	75.84±2.16	64.82±2.45*	65.38±1.82	66.37±2.36^{NS}
FS (%)	43.07±2.64	32.45±1.85*	32.8±1.6	33.98±1.57^{NS}
IVSd (mm)	0.93±0.04	1.17±0.06*	1.11±0.03	1.08±0.03^{NS}
LVIDd (mm)	3.82±0.1	4.06±0.13^{ns}	4.02±0.04	3.99±0.08^{NS}
LVPWd (mm)	1.08±0.07	1.25±0.07^{ns}	1.27±0.06	1.36±0.09^{NS}
HR (bpm)	462.8±8.24	482.5±4.25^{ns}	470.2±18.8	457.8±13.14^{NS}
HW/BW (mg/g)	5.62±0.14	6.78±0.33*	6.73±0.14	6.98±0.29^{NS}
HW/TL (mg/mm)	7.71±0.16	9.33±0.4*	9.09±0.31	8.99±0.33^{NS}

EF, ejection fraction; FS, fractional shortening; IVSd, diastole interventricular septum; LVIDd, diastole left ventricular internal diameter; LVPWd, diastole left ventricular posterior wall; HR, heart rate; HW, heart weight; BW, body weight; TL, tibial length; EV, empty vector; TAC, transverse aortic constriction. Data were shown as mean ± SEM. ns, $p>0.05$ vs SERCA2a^{WT}+EV+TAC; *, $p<0.05$ vs SERCA2a^{WT}+EV+TAC. NS, $p>0.05$ vs SERCA2a^{K628R}+EV+TAC.
